# Genome Dynamics Explain the Evolution of Flowering Time CCT Domain Gene Families in the Poaceae

**DOI:** 10.1371/journal.pone.0045307

**Published:** 2012-09-24

**Authors:** James Cockram, Thomas Thiel, Burkhard Steuernagel, Nils Stein, Stefan Taudien, Paul C. Bailey, Donal M. O'Sullivan

**Affiliations:** 1 John Bingham Laboratory, National Institute of Agricultural Botany, Huntington Road, Cambridge, United Kingdom; 2 Leibniz Institute for Plant Genetics and Crop Plant Research (IPK), Gatersleben, Germany; 3 Leibniz Institute for Age Research, Fritz Lipmann Institute (FLI), Jena, Germany; 4 Department of Computational and Systems Biology, John Innes Centre, Norwich, United Kingdom; Instituto de Biología Molecular y Celular de Plantas, Spain

## Abstract

Numerous CCT domain genes are known to control flowering in plants. They belong to the *CONSTANS*-like (*COL*) and *PREUDORESPONSE REGULATOR* (*PRR*) gene families, which in addition to a CCT domain possess B-box or response-regulator domains, respectively. *Ghd7* is the most recently identified *COL* gene to have a proven role in the control of flowering time in the Poaceae. However, as it lacks B-box domains, its inclusion within the *COL* gene family, technically, is incorrect. Here, we show *Ghd7* belongs to a larger family of previously uncharacterized Poaceae genes which possess just a single CCT domain, termed here *CCT MOTIF FAMILY* (*CMF*) genes. We molecularly describe the *CMF* (and related *COL* and *PRR*) gene families in four sequenced Poaceae species, as well as in the draft genome assembly of barley (*Hordeum vulgare*). Genetic mapping of the ten barley *CMF* genes identified, as well as twelve previously unmapped *HvCOL* and *HvPRR* genes, finds the majority map to colinear positions relative to their Poaceae orthologues. Combined inter-/intra-species comparative and phylogenetic analysis of *CMF, COL* and *PRR* gene families indicates they evolved prior to the monocot/dicot divergence ∼200 mya, with Poaceae *CMF* evolution described as the interplay between whole genome duplication in the ancestral cereal, and subsequent clade-specific mutation, deletion and duplication events. Given the proven role of *CMF* genes in the modulation of cereals flowering, the molecular, phylogenetic and comparative analysis of the Poaceae *CMF, COL* and *PRR* gene families presented here provides the foundation from which functional investigation can be undertaken.

## Introduction

The control of flowering time is a crucial environmental adaptation in plants, as well as a major determinant of grain yield in cereal crops [1]. Numerous environmental and endogenous signals combine via intricate molecular pathways to determine flowering time, with seasonal changes in photoperiod representing major cues for floral transition [2]. Studies of photoperiod pathway mutants in the long day (LD) photoperiod responsive dicot, arabidopsis (*Arabidopsis thaliana*), have shown the transcription factor *CONSTANS* (*CO*) promotes flowering under LDs [3]. *CO* is an output of the circadian clock, showing circadian oscillations of expression regulated by the circadian clock and light-induced degradation, such that coincidence of peak expression with light only occurs under LD photoperiods [4]–[5]. Light-stabilized CO induces transcription of downstream genes such as the phytohormone *FLOWERING LOCUS T* (*FT*), which controls vegetative-to-reproductive transitioning of the shoot apical meristem by integrating inputs from various pathways [6]–[8]. The photoperiodic pathway is highly conserved within the plant kingdom, from the unicellular green alga *Chlamydomonas reinhardtii* [9] to tree species such as *Populus tremula* [10]. The observation that green micro-algae, but not earlier photosynthetic microorganisms such as diatoms and euglenoids, possess *CO-*like (*COL*) genes suggests that the gene family appeared during or soon after the endosymbiotic event in the photosynthetic lineage [5] and has evolved to regulate flowering in response to inductive daylengths in higher plants. In arabidopsis, *CO* belongs to a larger family of 17 *COL* genes, subdivided into three broad subgroups [11]. They encode proteins with a conserved region of 43 amino acids towards their carboxy-terminus, termed the CCT domain, which in arabidopsis interacts with CONSTITUITIVE PHOTOMORPHOGENIC 1 (COP1) protein to control CO stability [12]. In addition to the CCT domain, COL proteins contain one or two zinc-finger B-box domains towards the amino terminus, thought to be involved in protein-protein interactions [5]. While *COL* genes have been identified in various plant species [11], [13]–[14], comparatively little is known about their function. As well as *COL* genes, an additional CCT domain gene family has been shown to play a role in the photoperiod pathway. Termed *PSEUDO RESPONSE REGULATORs* (*PRRs*), they contain a response regulator domain towards the amino-terminus, as well as a CCT domain at the carboxy-terminus. *PRR* genes were first identified in arabidopsis, where *PRR1* (*TOC1*) acts within the central oscillator of the circadian clock [15]. Although five *PRR* genes are found in arabidopsis, the precise function of the remaining four genes has not been determined [16]. *PRR* genes are also found in the Poaceae, with rice (*Oryza sativa* L.), barley (*Hordeum vulgare* ssp. *vulgare* L.) and sorghum (*Sorghum bicolour* L. Moench) all possessing five members [17]–[20].

In cereals, map-based cloning shows that natural variation within CCT domain genes have been critical in the control of flowering and spread of cereal domestication. Orthologous Poaceae *PRR* genes are known to encode LD photoperiod responsive loci in barley (*PPD-H1*, encoded by *HvPRR37*), sorghum (*Ma1*, encoded by *SiPRR37*) and hexaploid bread wheat *Triticum aestivum* L. (*PPD-D1*, encoded by *TaPRR37-D*) [18], [20]–[21]. Recessive alleles at all loci result in a delay in flowering under LDs, and have aided their spread of domestication into new agricultural environments. Furthermore, *OsPRR37* has been mapped to the rice *Heading date 2* (*Hd2*) quantitative trait locus (QTL) interval, with the photoperiod insensitive Kasalath allele associated with a severe mutational lesion in *OsPRR37* [17]. Similarly, *COL* genes have been shown to control natural variation in flowering time in rice, where *Heading date1* (*Hd1*, homologous to *CO*) induces the transcription of *Hd3a* (homologous to *FT*) promoting flowering under short day (SD) photoperiods [22]–[24]. Genes belonging to a Poaceae-specific diverged *COL* subgroup, previously termed the group IV *COL* genes [13], have been shown to control flowering time in temperate cereal species. The first to be identified were the *ZCCT* genes that underlie colinear *VRN-2* vernalization response loci in temperate cereals. Their predicted proteins encode a CCT domain and a putative single amino zinc finger, divergent from the B-box domains found in other *COL* genes [25]. In the diploid wheat *T. monococcum*, the *VRN-A^m^2* locus was found to be encoded by *VRN2* (also termed *ZCCT1*, and used hereafter) [26]. Subsequently, orthologous *ZCCT* genes were found to co-segregate with the *VRN-H2* locus in barley [27] and the *VRN-A1/VRN-B1* loci in the tetraploid wheat, *T. turgidum* [28]. Recessive mutant alleles at colinear cereal *VRN-2* genetic loci abolish vernalization response, resulting in spring-sown crop ideotypes that allowed flexibility of cultivation and adaptation to new agricultural environments. In spring barley, the three *ZCCT-H* genes underlying the *VRN-H2* locus have been deleted in all accessions investigated to date [29]–[32]. Similarly, *ZCCT* copy number variation is associated with flowering time in tetraploid wheat [28]. Interestingly, no *ZCCT* genes are present at the colinear regions of the SD tropical plant rice, or in the sequenced rapid cycling accession of brachypodium [33]. Recently, map-based cloning of the rice flowering time locus *Ghd7* (previously termed, *OsI* [13]) has found it to be encoded by a circadianly regulated group IV *COL* gene [34]. Enhanced expression of *Ghd7* under non-inductive LD photoperiods delays flowering. Furthermore, naturally occurring *Ghd7* deletions/mutations alleviate floral repression under LDs. Thus, differential retention/deletion/mutation of genes containing CCT domains appears to have been critical in the domestication and adaptation of cereal crops. The Triticeae *ZCCT* genes, along with *Ghd7* and cereal orthologues of the rice gene *OsH* form a distinct grouping within the *COL* genes [26], [28]–[29]. By comparing inter- and intra-species microcolinearity between rice, brachypodium and barley, we previously showed that the *ZCCT* genes and *OsH* are evolutionarily related [33], forming a paralogous gene-pair that arose during the whole genome duplication event that occurred in the ancestral cereal genome [35]–[36].

While sixteen *COL* genes (*OsA-OsP*) have been identified in rice [13], the total should strictly be reduced to fourteen, due to the lack of B-box domains in *OsH* and *Ghd7*. Recently, eight members homologous to arabidopsis *Activator of Spo^min^::LUC2* (*ASML2*) have been identified, which also encode proteins possessing just a single CCT domain [37]. However, no systematic investigation of this class of gene has been undertaken in any Poaceae species, or indeed, in arabidopsis. Preliminary investigation in rice shows that such genes are relatively common, with thirteen members (including *OsH* and *Ghd7*) identifiable in the current genome assembly. Accordingly, we name this group of genes the ‘*CCT MOTIF FAMILY*’ (*CMF*), following the protein annotation in the rice genome assembly. Thus, while analyses of *COL* or *PRR* genes and gene families have been previously undertaken to varying extents in barley, rice, sorghum and brachypodium [13], [19]–[20], comprehensive analysis of all CCT domain gene families known to possess members that regulate flowering in the Poaceae, and specifically the largely unrecognised *CMF* gene family, is yet to be undertaken in the grasses. To determine if *CMF* genes arose independently by species-specific 5′ truncation of *COL* or *PRR* genes, or whether they represent a more ancient gene family, we undertake molecular, phylogenetic and comparative genomic analysis of the *CMF, COL* and *PRR* gene families in sequenced Poaceae genomes, as well the crop species barley, which lacks a sequenced physical map. Finally, we propose a systematic nomenclature for Poaceae *CMF* genes, based on orthology between species.

## Materials and Methods

### Bioinformatic analysis

*CMF, COL* and *PRR* gene families were determined in the current rice genome assembly (*Oryza sativa* ssp. japonica cv. Nipponbare, MSU Osa1 assembly v6.1, http://rice.plantbiology.msu.edu/) using BLASTn searches (match / mismatch scores = 2,3; gap costs: existence = 5, extension = 2) and an expectation (e)-value threshold of 1.0e-30. For the *COL* family, coding regions (CDS) for each of the fourteen known rice genes [13] were used as queries, while for the *PRR* family, CDS of the five genes listed by [18] were used. *CMF* genes were identified in rice and arabidopsis (TAIR annotation release 10 of the *A. thaliana* genome, release 9) by BLAST analysis of CDS from *OsH, OsI* [13] and arabidopsis homologues [37]. Unless otherwise stated, primary transcripts were used throughout. Predicted proteins were translated using the VectorNTI Advance package v10.1.1 (Invitrogen) and protein domains determined using Pfam v25.0 [38] and Prosite v20.79 (http://prosite.expasy.org/). Amino acid sequence representation was undertaken using WebLogo [39]. Proteins identified as containing a CCT motif (and lacking additional domains) were annotated as *CMF* genes. Proteins additionally containing B-box and pseudoresponse regulator domains towards their N-termini were classified as *COL* or *PRR* genes, respectively. Following the approaches listed above, *COL, PRR* and *CMF* gene families were determined in *B. distachyon* accession BD21 (assembly v1.0, using sequence data produced by the US Department of Energy Joint Genome Institute, http://modelcop.org/), *S. bicolour* L. Moench accession BTx623 (v1.0, http://phytozome.net/), and *Setaria italica* cv. Yugu1 (foxtail millet assembly v1.0, using sequence data produced by the US Department of Energy Joint Genome Institute, http://phytozome.net/). Barley *CMF, COL* and *PRR* sequences were identified by BLASTn searches of the rice CDS versus a 28× NGS genomic survey of cv. Morex (generated by the International Barley Sequencing Consortium, available at http://mips.helmholtz-muenchen.de/plant/triticeae/) or fl-cDNAs [40]. *De novo* gene predictions and reassessments were conducted using FGENESH (http://www.softberry.com/) using ‘monocot’ as the basis of gene prediction. Homology groupings between *CMF*, *COL* and *PRR* genes identified in brachypodium, sorghum, foxtail millet and barley were verified by back-BLASTn searches of the rice genome, to ensure highest sequence similarity to the original rice query sequence. Genes are prefixed with the genus and species initials.

### Phylogenetic analysis

CCT domain protein sequences were aligned using ClustalW [42] and manually edited using GENEDOC v2.6 (http://www.nrbsc.org/gfx/genedoc/). Phylogenetic analysis was conducted on the resulting alignments using the PHYLIP package v3.5 [43]. Unrooted phylogenies were determined using the distance matrix method, with tree topographies supported by bootstrapping (1,000 replicates).

### Genetic mapping

Genomic DNA was extracted from leaf material using the DNeasy 96 Plant Kit (Qiagen). Genetic mapping was undertaken in the barley OWB doubled haploid population [44]. Primers for SNP identification in parental lines and genetic mapping in the complete population were designed using Primer3 v0.4.0 (http://primer3.sourceforge.net/) (Table 1). PCR amplification was carried out in 10 µl reactions using the reagents shipped in the FastStart *Taq* DNA polymerase kit (Roche). PCR cycling was carried out using a Veriti 96 well Thermo Cycler Thermocycler (Applied Biosystems) with the parameters: 5 min at 96°C, followed by 35 cycles of 50 sec at 96°C, 50 sec annealing temperature, 90 sec at 72°C, final extension of 7 min at 72°C. Annealing temperatures are listed in Table 1. Sequencing in parental lines used a minimum of three independent PCRs as templates for sequencing using BigDye kit v3.1 (Applied Biosystems), following the protocols described by [45]. Sequence traces were manipulated using VectorNTI. DNA polymorphisms were genotyped in the mapping population by direct sequencing of PCR amplicons using the polymorphisms listed in Table 1. Genetic mapping was conducted using JoinMap v3.0 [46]. Genetic map positions of *COL, CMF*, *PRR* and *ZCCT* genes in the OWB population were integrated into the barley consensus map [47] based on co-segregation with genetic markers common to both maps. Genomic nucleotide sequences derived from OWB-D and OWB-R parental lines have been deposited in GenBank under accession numbers JQ791213–JQ791252.

**Table 1:** Genetic mapping of barley genes.

Gene	Rice orthologue	Primer sequence (5′→3′)^1^	A/GC^2^	GenBank accession^3^	Mapped SNP	Chromosome (cM)^4^
*HvCO10*	Os03g50310	F: **CACCTCCTCCCTTCAGCAC**	60°C/+	D: JQ791236	G68/T	4Hs (32.5)
		R: CGTATCTTCTTGGCGAACAG		R: JQ791237		
*HvCO11*	Os02g49880	F: **CGTTCTACATGGAGGCCCTA**	60°C/+	D: JQ791238	G93/A	6Hl (93.1)
		R: TTCTGTTCCCGGTCATTCTC		R: JQ791239		
*HvCO12*	Os06g15330	F: AGCACAAGTGTGACGTGGAG	60°C/+	D: JQ791240	A285/G	7H (74.1)
		R: **GATGCACCATACCGTGACTG**		R: JQ791241		
*HvCO13*	Os06g19444	F: CTTCCGAAGCTGGTCTGAAC	58°C/+	D: JQ791242	G300/T	7H (74.1)
		R: **TGTTGCATATGCCAGTCCAT**		R: JQ791243		
*HvCO14*	Os02g49230	F: GCCGCATCTAGCTTTTTGTC	60°C/+	D: JQ791244	C3824/T	6Hl (89.7)
		R: **CCACGTTTTGAGTTGTGGT**		R: JQ791245		
*HvCO15*	Os08g42440^5^	F: **TTCTCCGATCGGTTTTTCAC**	60°C/+	D: JQ791246	T92/G	7H (80.7)
		R: AGCTGAGAGGCCTACCACAA		R: JQ791247		
*HvCO16*	Os03g22770	F: **CATGGAGAAGCCGTTCTTGT**	60°C/+	D: JQ791248	A128/G	4Hl (54.4)
		R: TTGCAACCTCATGGCTGTTA		R: JQ791249		
*HvCO18*	Os07g47140	F: CAACAACAAGGTGGACGAGA	58°C/+	D: JQ791250	C640/G	2Hs (55.2)
		R: **CAAGATCAGCATGCCCAGTA**		R: JQ791251		
*HvCMF1*	Os01g61900	F: GACCCATCCATCGCCTACTA	60°C/−	D: JQ791213	C340/G	3Hl (127.9)
		R: **GCATTCTTCTCGTCCTCGTC**		R: JQ791214		
*HvCMF3*	Os02g05470	F: GGCTTTTCTGCAATTTGCTC	60°C/−	D: JQ791215	A232/T	6Hs (60.9)
		R: **CAGCGAGGCTTAGTGAATCC**		R: JQ791216		
*HvCMF4*	Os03g04620	F: CAATGCAAATGAGCAGCACT	60°C/+	D: JQ791217	A148/T	4Hl (101.9)
		R: **TCAGCTGAAAGACGCACAAC**		R: JQ791218		
*HvCMF5*	Os05g38990	F: GAGGTCGTTGTATGGGCAGT	60°C/−	D: N/A	InDel	1Hl (94.2)
		R: CAGACACACCAGAGGGGATT		R: JQ791219		
*HvCMF6a*	Os05g51690	F: CATAGTCGAGGAACCGCTG	60°C/−	D: JQ791220	In322/Del	1Hl (156.3)
		R: **TTCTAGACGCAGACAAGCCA**		R: JQ791221		
*HvCMF6b*	Os05g51690	F: CATAGTCGAGGAACCGCTG	59°C/+	D: JQ791252	InDel	1Hl (156.3)
		R: CACTCGTAGACTCTATGCTCTCA		R: N/A		
*HvCMF7*	Os06g48610	F: GGAAATGCTTCTTTGGGTGA	58°C/−	D: JQ791222	T505/C	7Hl (114.6)
		R: **AGCCGTCATCTGCTTCACTT**		R: JQ791223		
*HvCMF10*	Os10g32900	F: **GCGGACCCATTGTAAAAGAA**	60°C/−	D: JQ791224	T1155/A	1H (57.7)
		R: AGAGTGGGTAGGGTGGCTTT		R: JQ791225		
*HvCMF13*	Os12g01080	F: **CATCCATCCTGCCTTCATCT**	58°C/+	D: JQ791226	T635/C	5Hl (64.9)
		R: AGCGCAACTTCCAAAAGAAA		R: JQ791227		
*HvPRR59*	Os11g05930	F: **GAGGCGCCACTCTGTAAGTC**	60°C/+	D: JQ791228	A143/G	4Hl (52.3)
		R: AGAACGTTGCTGCTTCCCTA		R: JQ791229		
*HvPRR73*	Os03g17570	F: **CTTCAGACCCAGCTCTTTGG**	60°C/+	D: JQ791230	G1104/A	4Hl (54.4)
		R: AGCCCACAAAACCCACTATG		R: JQ791231		
*HvPRR95*	Os09g36220	F: **CTCAGGGAAAGCTCCAACTG**	60°C/+	D: JQ791232	T983/A	5Hl (131.5)
		R: GTGTCAAGGCCTGGGAATTA		R: JQ791233		
*HvTOC1*	Os02g40510	F: **TGAAGTGCCATGCTATGCTC**	60°C/+	D: JQ791234	G936/T	6Hl (68.5)
		R: CGAAGGAGACGGATGGTAAA		R: JQ791235		

1 Forward (F) and reverse (R) primer; primers highlighted in bold were used for direct sequencing for detection of polymorphisms.

2 A/GC = anealing temperature/GC buffer (+ = GC buffer added, − = no GC buffer added).

3 GenBank accessions for genomic sequences from OWB-D (D) and OWB-R (R) parental lines are indicated. SNP positions are relative to OWB-D.

4 Barley chromosome arm: short (s), long (l). *HvCMF5* and *HvCMF6b* were genetically mapped using presence/absence PCR/agarose gel InDel assays.

5 Due to lack of polymorphism in the genomic fragment investigated, *HvCO15* was mapped using a SNP in an adjacent predicted gene (*HvOs08g42430*) located on the same sequence contig (Table S4).

### Poaceae colinearity and nomenclature

Colinearity between rice (Mbp) and the barley consensus genetic map (cM) was determined as described by [48]. Rice chromosomal regions duplicated during the ancestral WGD were identified in the current rice genome assembly as previously described [49], and plotted using Circos [50]. Microcolinearity between orthologous Poaceae *CMF* genes was determined using ±10 genes on each side of each *OsCMF* gene for BLASTn query of the sequenced genomes of brachypodium, sorghum and foxtail millet, using the BLAST parameters listed above. Poaceae macro-colinearity follows that previously described [51]–[52]. Previously mapped *COL, PRR* and *ZCCT* genes [13], [18], [29] are integrated into the barley consensus map [47] based on common markers and established inter-specific barley-rice colinearity. For clarity when comparing orthologous genetic loci and genes, we use the vernalization locus nomenclature described by [41], and *ZCCT1* (also called *VRN2*) to denote the gene underlying the *VRN-A^m^2* locus in *T. monococcum*. All gene synonyms are listed in Tables S1–S5. The updated Poaceae *COL* nomenclature presented here builds on the numerical numbering system previously introduced for brachypodium, sorghum and barley in these species. However, to avoid confusion, the reclassification of *HvCO9* as *CMF11* (due to lack of B-boxes) has meant we have had to miss out *CO9* in the otherwise consecutive ordering of Poaceae *CO* genes identified here. Rice *COL* nomenclature continues the alphabetical system previously used [13].

## Results

Using CDS from the eight previously reported arabidopsis *CMF* genes [37] a total of fifteen arabidopsis family members were identified (Table S1). Towards understanding the evolution of Poaceae CCT domains genes, we then determined the *CMF, COL, PRR* and *ZCCT* gene families in the sequenced genomes of rice, brachypodium, sorghum, foxtail millet, as well as in the preliminary draft barley genome. Results are summarised in Tables S2–S5.

### Poaceae *COL* genes

With the recognition of the *CMF* gene family, the rice *COL* gene family [13] should be reduced from sixteen (*OsA-OsP*) to fourteen members, due to the lack of B-box domains in *OsH* and *Ghd7* (*OsI*). To determine *COL* copy number in the current rice genome assembly, we used *OsCOL* CDS for BLASTn analyses. Three additional *OsCOL* genes were identified (termed *OsQ, OsR* and *OsS*, respectively), resulting in a total of seventeen *COL* genes, distributed across chromosomes Os2–Os4 and Os6–Os9 (Table S2). All three novel *OsCOL* genes were predicted to possess one B-box domain, located towards the N-terminus of their predicted proteins. Analysis of the brachypodium genome returned sixteen *COL* genes. No homologues *OsB* and *OsQ* were identified, although one *BdCOL* gene lacking a rice homologue rice was identified (*BdCO2* [19]). Sorghum and foxtail millet were fond to possess sixteen and seventeen *COL* genes, respectively. As well as lacking homologues of *OsB* and *OsQ*, both species possessed a single additional *COL* gene (*SbCO20* and *SiCO20*), absent in the temperate grasses investigated here. Similarly, sorghum and foxtail millet contained homologues of *BdCO2*, although in sorghum, the predicted protein did not contain a B-box domain, and is therefore classified as a *CMF* gene (*SbCMF16*). Three of the foxtail millet *COL* genes (*SiCO1, SiCO5* and *SiCO19*) were identified by gene prediction reanalysis of genomic regions identified by BLASTn analysis. In addition to the previously identified barley orthologues of *OsA* (*HvCO1*), *OsB-OsG* (*HvCO3-HvCO8*) and *BdCO2* (*HvCO2*) [13], [19], searches of the preliminary draft barley genome and full length (fl) cDNAs identified eight additional full-length *COL* genes homologous to *OsJ-OsP* and *OsR*. Barley *OsL-OsN* homologues were identified on genomic contigs of size 4,069–6,808 bp, with gene prediction software identifying just one gene per contig. Remaining *HvCOL* genes were identified from fl-cDNA sequences. No barley homologues were identified for *OsQ* and *OsS*.

### Poaceae *PRR* genes

Five *PRR* genes were identified in the genomes of rice and brachypodium (Table S3), agreeing with previous reports [18]–[19]. Similarly, five *PRR* genes were found in foxtail millet (chromosomes Si1–2, Si8–9) and sorghum (chromosomes Sb1–2, Sb4–6). Although no sorghum gene models homologous to *OsPRR95* were identified, an unannotated genomic region with high sequence homology (4.3e-60) was identified. Closer inspection found a sequencing gap spanning predicted *SbPRR95* exon 6 and part of exon 7, indicating complete sequencing would resolve an *SbPRR95* gene model. For *SbPRR37*, the single annotated splice transcript contains seven exons. However, it should more correctly be annotated according to the photoperiod insensitive *sbprr37-2 ma1* allele, previously reported to possess 8 exons [20]. Searches in barley identified five *PRR* genes, including the barley *OsPRR37* orthologue that encodes the photoperiod locus *PPD-H1* [18]. Three of the remaining four *HvPRR* genes were predicted to encode full length CDS, while lack of available genomic sequence corresponding to *HvPRR59* means that the resulting predicted protein is truncated upstream of exon 2.

### Poaceae *CMF* genes

Using CDS for *OsH* and *Ghd7* (*OsI*) (both previously grouped within the *COL* gene family) and the arabidopsis *CMF* genes, an additional twelve members of the rice *CMF* gene family were identified, resulting in fourteen members distributed between ten chromosomes (Table S4). All contained a single CCT domain as the only identifiable domain within their predicted proteins, and are named here in chromosome order as *OsCMF1-OsCMF14*. In all but three *CMF* genes (*OsCMF4, OsCMF10, OsCMF11*), the CCT domain is encoded within regions spanning the penultimate and last exon (Figure S1). *OsCMF* physical map locations show they are located in distinct genomic regions relative to *OsCOL* genes (Table S2,S4), indicating the two gene families did not arise due to tandem duplication and subsequent loss of B-box domains in the rice lineage. Brachypodium was found to contain eleven *CMF* members. While homologues for *OsCMF2, OsCMF8, OsCMF12* and *OsCMF14* are absent, one additional brachypodium member was identified: *BdCMF15* (for which no homologues were identified in any of the other Poaceae species investigated). *CMF* homologues for twelve of the fourteen rice members were identified in sorghum, with CDS ranging from 678–1374 bp. Foxtail millet was found to possess eleven *CMF* genes, lacking homologues of rice genes *OsCMF2, OsCMF13* and *OsCMF14*. Although three *SiCMF* genes were not annotated as *S. italica* gene models, gene-prediction reanalysis of genomic regions identified by BLASTn analysis resolved *CMF* genes in all three instances (Table S4). Barley *CMF* genes were identified for nine of the thirteen rice members (*HvCMF1, HvCMF3-HvCMF7, HvCMF10–11, HvCMF13*), with no barley homologues of *OsCMF2, OsCMF8, OsCMF9, OsCMF12* and *OsCMF14* identified. All but two of these (*HvCMF1* and *HvCMF3*) were predicted to encode full length transcripts. Two fl-cDNA sequences with high homology to *OsCMF6* were identified, and are named *HvCMF6a* (AK250075) and *HvCMF6b* (AK355694). Finally, previous studies show that while the rice paralogue of *OsH* (chromosome Os10) has been lost on chromosome Os03 after whole genome duplication (WGD) in the ancestral cereal genome, the paralogous gene has been retained on the long arm of the temperate cereal group 4 chromosomes [33]. In barley, three cosegregating copies of the ancestral paralogue (*ZCCT-Ha, ZCCT-Hb* and *ZCCT–Hc*) are present in vernalization sensitive varieties [30]. As expected, BLASTn analysis of the vernalization insensitive barley varieties ‘Morex’ and ‘Haruna Nijo’ did not return any Z*CCT-H* genes. Accordingly, previously published sequences identified in vernalization sensitive lines [29] are used in subsequent molecular and phylogenetic analyses.

### CMF, COL, PRR and ZCCT protein domain and coding region analysis

**Figure 1:**
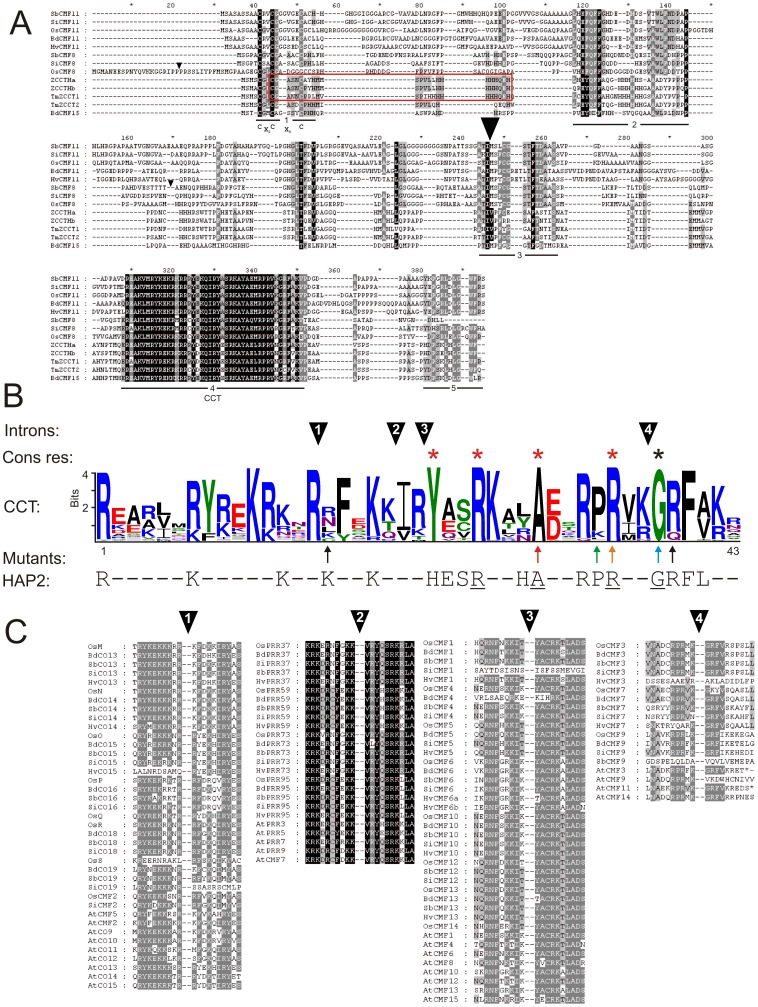
Protein domain analysis. Intron positions are indicated by triangles. (a) Alignment of CMF8, CMF11, ZCCT and BdCMF14 proteins. Conserved regions are underlined. The Zinc finger is boxed in red, and the conserved residues present at the N-terminus end of Poaceae COL B-box1 are indicated. (b) WebLogo representation of CCT domains from Poaceae CMF, COL, PRR and ZCCT sequences. Residues conserved in all proteins, and all proteins except HvCMF3, are indicated by red and black asterisks, respectively. Arrows indicate the position of mutated CCT domain residues in *T. monococcum* ZCCT1 (TmZCCT1, black), *Arabidopsis thaliana* CO (AtCO, green), PPD-H1 (blue), AtCO and ZCCT1 (orange), AtCO and AtTOC1 (red) [11], [18], [26], [59]. HAP2 amino acid sequence conserved with the Poaceae CCT domains is illustrated, and common residues underlined. (c) Conservation of intron/exon boundaries in Poaceae *CMF, COL* and *PRR* genes which possess an intron within the CCT domain.

COL proteins possess either one or two B-boxes, in addition to a CCT domain. All Poaceae COL proteins identified here possess protein domain configurations identical to their closest rice homologues (Table S2). The three novel rice COL proteins (*OsR-OsS*) and their Poaceae homologues all possess a single B-box, while SbCO20/SiCO20 and BdCO2 homologues in sorghum and foxtail millet contain two B-boxes. Three of the four *ZCCT* genes investigated (*ZCCT1, ZCCT-Ha, ZCCT-Hb*) were predicted to encode zinc-finger domains towards the N-terminus, as previously reported [26], [29], which shows some similarity to the conserved C and H residues of COL B-box1 (Figure 1a). Alignment of CCT domains from Poaceae CMF, COL, PRR and ZCCT CCT proteins investigated in subsequent phylogenetic analysis, finds perfect amino acid conservation at four positions: Tyr^23^-Arg^26^-Ala^30^-Arg^35^ (Figure 1b). In addition, Gly^38^ was conserved in the proteins of all but one (*HvCMF3*) of the Poaceae genes investigated. Excluding the CCT domain, Poaceae CMF proteins did not share additional common conserved regions, nor were further protein domains identified. However, dispersed regions of conservation were evident between smaller sub-groups of CMF proteins (Figure 1a, Figure S2). Exon numbers for homologous genes commonly varied between species, although nine *COL* (homologues of rice genes *OsA,OsC-OsD,OsG,OsJ-OsN*), three *CMF* (*CMF1,CMF5,CMF11*) and three *PRR* (*PRR59,PRR73,TOC1*) genes shared identical numbers in all four of the sequenced Poaceae species investigated (Table S2–S4). To explore gene family structure and evolution in more detail, protein sequences immediately adjacent to introns bisecting CCT domains were aligned, resolving four major groups (Figure 1c). The first contains proteins with an intron after CCT residue 15, and is composed predominantly of *COL* genes (homologues of rice genes *OsM-OsS*), along with four *CMF* genes (*OsCMF2, SiCMF2, AtCMF2, AtCMF5*). The second group possess an intron after residue 20, and contains Poaceae *PRR* genes (excluding *TOC1* homologues, which lack a CCT domain intron), as well as *AtCMF7*. Group 3 has an intron located after residue 22, and consists exclusively of Poaceae and arabidopsis *CMF* genes (*CMF1, CMF4-CMF6, CMF10, CMF12-CMF14*). Similarly, the final group exclusively contains *CMF* genes from the Poaceae (*CMF3, CMF7, CMF9*) and arabidopsis *CMF* (*AtCMF3, AtCMF9, AtCMF11, AtCMF14*), which all possess an intron after residue 37. While all remaining genes lack introns within the CCT domain (*CMF8, CMF11, ZCCT, BdCMF15*), alignment of their protein sequences find all to possess an intron at a conserved position, distinct from those observed in other *COL* genes (Figure S3,S4), with additional regions of conservation identified across their peptides (Figure S1).

### Phylogenetic analysis of Poaceae *CMF*, *COL, PRR* and *ZCCT* gene families

**Figure 2:**
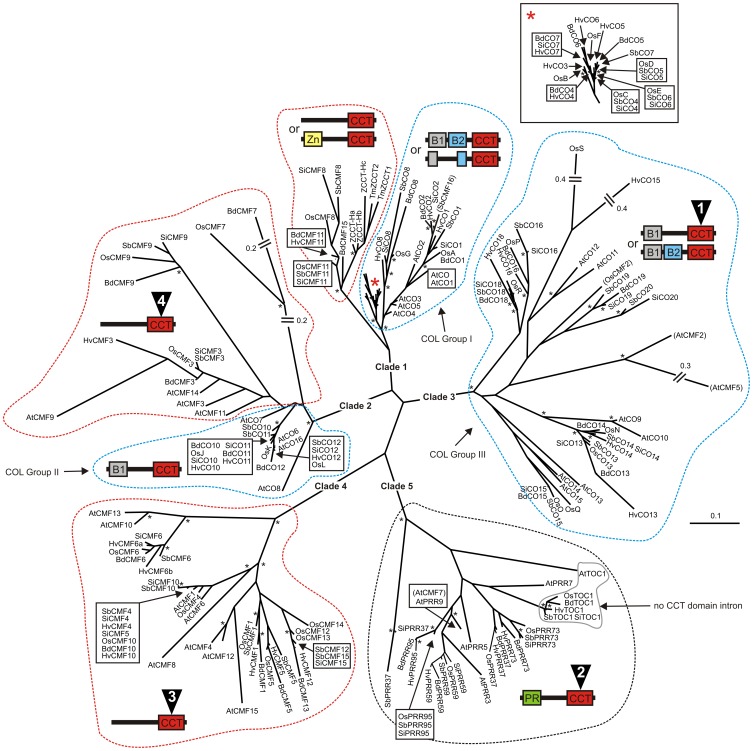
Phylogenetic analysis of CMF, COL, PRR and ZCCT proteins, based on the CCT domain. Dashed lines enclose predominantly CMF (red), COL (blue) and PRR (black) families, within which the predominant protein domain configurations within full length proteins are indicated (proteins lacking the predominant pattern are indicated in brackets). Numbered triangles indicate CCT domain intron position (Figure 1). COL clades [following 11,13] are indicated with COL I and II proteins possessing two or one B-box domains, respectively. Genes are prefixed with genus and species initials. Gene synonyms as listed in Supplementary Tables S1–S5. Although Group III *COL* genes are previously described as posessing two B-boxes, domain prediction software used here identified variously one or two B-boxes. Arabidopsis CMF (Table S1), COL [13] and PRR [19] members are included, as are ZCCT1 (also termed VRN2, AAS60241) and ZCCT2 (AAS60248) from *T. monococcum*. Black asterisks indicate nodes with bootstrap values >60%. SiCMF1, BdCMF4, HvCMF7, SbCMF7 and SiCMF7 are excluded, due to deletions within the CCT domain.

To help understand the evolution of the *CMF* gene family, protein sequence from the CCT domains encoded by Poaceae and arabidopsis *CMF, COL, PRR* and *ZCCT* genes were used to construct a Neighbour-Joining phylogenetic tree. Analysis resolved five major groupings, named Clade 1–5 (Figure 2). All Poaceae *PRR* genes clustered together within Clade 5, along with a single arabidopsis *CMF* gene (*AtCMF7*), indicating it may represent a recently truncated *PRR* gene. All Poaceae *CMF, COL* and *ZCCT* genes were partitioned within the four remaining phylogenetic groupings. Clade 4 compromised exclusively of Poaceae and arabidopsis *CMF* genes, with sub-clustering evident for gene pairs *CMF1-CMF5, DMF4*-*CMF10* and *CMF12-CMF13*. Poaceae *CMF6* genes formed a distinct sub-cluster within Clade 4, with the CCT domain encoded by *HvCMF6b* the most diverged. In addition, eight of the fifteen arabidopsis *CMF* homologues clustered within Clade 4. All but three of the forty-two Clade 3 genes belong to the *COL* gene family, and possess a single zinc finger domain towards their N-terminus. This class of rice gene has previously been termed the *COL* Group III gene family (*OsM-OsP*) [13], and also includes the three additional Poaceae *COL* genes identified in this study (homologous to rice genes *OsQ-OsS*). Sub-clustering of Poaceae *COL* gene pairs homologous to *OsM-OsN, OsO-OsQ, OsP-OsR* and *OsS-OsT* was evident, while arabidopsis genes *AtCMF2* and *AtCMF5* formed a distinct grouping off the *S-T* branch, as did a single *CMF* gene (*OsCMF2*). No *OsCMF2* homologues were identified in any other species investigated, suggesting it represents a recently truncated *COL* gene. Clade 2 consists of two separate sub-clades. The first is made up of the Poaceae and arabidopsis Group II *COL* genes, while the second consists of a cluster of Poaceae *CMF3* and *CMF9* members, four arabidopsis *CMF* genes (*AtCMF3,9,11,14*), and the Poaceae *CMF7* genes (*SbCMF7, SiCMF7* and *HvCMF7* were excluded from phylogenetic analysis due to deletions within the genomic region encoding the CCT domain). Clade 1 consists of two sub-groupings, and with the exception of *SbCMF16*, the first consists of the rice Group I rice *COL* genes (*OsA-OsG*) and their Poaceae homologues, with clustering of gene pairs *OsC-OsD* and *OsE-OsF*. The second sub-grouping contains fourteen *CMF* genes split between two main branches. The first branch contains *CMF11* homologues from each of the five Poaceae species investigated, as well as *CMF8* genes from rice, sorghum and foxtail millet. The first member to split from the base of the second branch is *BdCMF15*, for which no homologues were identified in any other species. The remainder of the branch consists exclusively of the barley *ZCCT-Ha, -Hb, -Hc* genes that underlie the *VRN-H2* flowering time genetic locus, as well as the orthologous *ZCCT1* and *ZCCT2* genes, located at the colinear *VRN-A^m^2* locus in *T. monococcum* (detailed in Table S5).

### Genetic mapping of barley *CMF*, *COL* and *PRR* genes

As nine *COL* genes and *HvPRR37* (*PPD-H1*) have previously been genetically mapped [13], [18], their map locations in the barley consensus map are estimated utilizing comparative approaches (Text S1). Of the additional eight *HvCOL* and four *PRR* genes identified here, all were found to contain genetic polymorphisms between parental Oregon Wolfe Barley-Dominant (OWB-D) and OWB-Recessive (OWB-R) lines, allowing subsequent genetic mapping (Table 1, Text S2). Similarly, polymorphisms identified in all ten barley *CMF* genes allowed all to be genetically mapped. Using SNP C340/G (Gln → Glu), *HvCMF1* was mapped to the long arm of chromosome 1H at 127.9 cM, between SNP markers 11_21081 and 11_21277. Using a SNP in the 3′UTR *HvCMF3* was found to cosegregate with 17 genetic markers at 60.9 cM on 6H, while *HvCMF4* cosegregated with four genetic markers at 101.9 cM on the long arm of 4H. A presence/absence polymorphism mapped *HvCMF5* to 94.2 cM on the long arm of 1H, cosegregating with 12_30072 and bPb-8477. Two *OsCMF6* homologues were identified in barley: an 11 bp intronic InDel allowed mapping of *HvCMF6a* to the distal end of chromosome 1H at 156.3 cM, where it cosegregated with nine previously mapped markers. *HvCMF6b* was found to cosegregate with *HvCMF6a*, using a presence (OWB-D)/absence (OWB-R) polymorphism. Intronic SNP T505/C allowed *HvCMF7* to be mapped to 114.6 cM, on the long arm of 7H. Polymorphisms downstream of predicted stop codons were used for the remaining *HvCMF* genes, allowing *HvCMF10* and *HvCMF13* to be mapped to chromosome 1H (57.7 cM) and 5H (64.9 cM), respectively. We previously mapped *HvCMF11* (previously known as *HvCO9*) to the long arm of 1H, were it was found to cosegregate with five gene-based markers [33], allowing integration into the consensus map (Text S1).

### Comparative genomic analysis of Poaceae *CMF*, *COL* and *PRR* gene families

**Figure 3:**
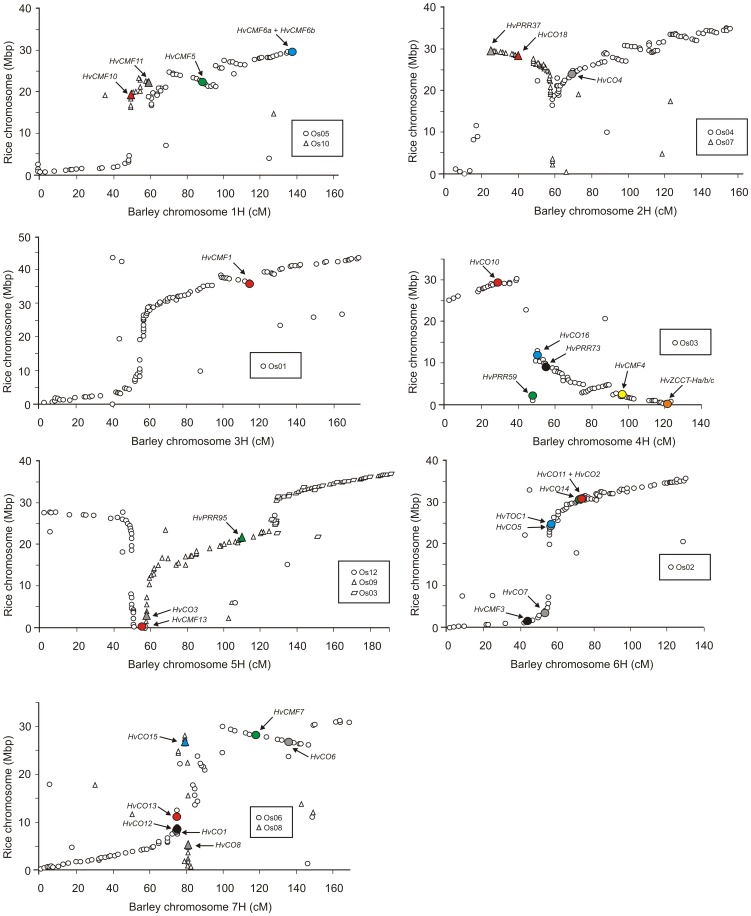
Barley-rice colinearity, indicating CCT domain genes. The position of barley gene-based genetic markers in the barley consensus genetic map [47] are indicated on the x-axis (cM). The locations of their orthologues in the rice physical map are indicated on the y-axis (Mbp). Genes mapped in the OWB population, as well as previously mapped genes included within the figure (highlighted in grey), are integrated into the consensus genetic map as described in Text S1,S3.

**Figure 4:**
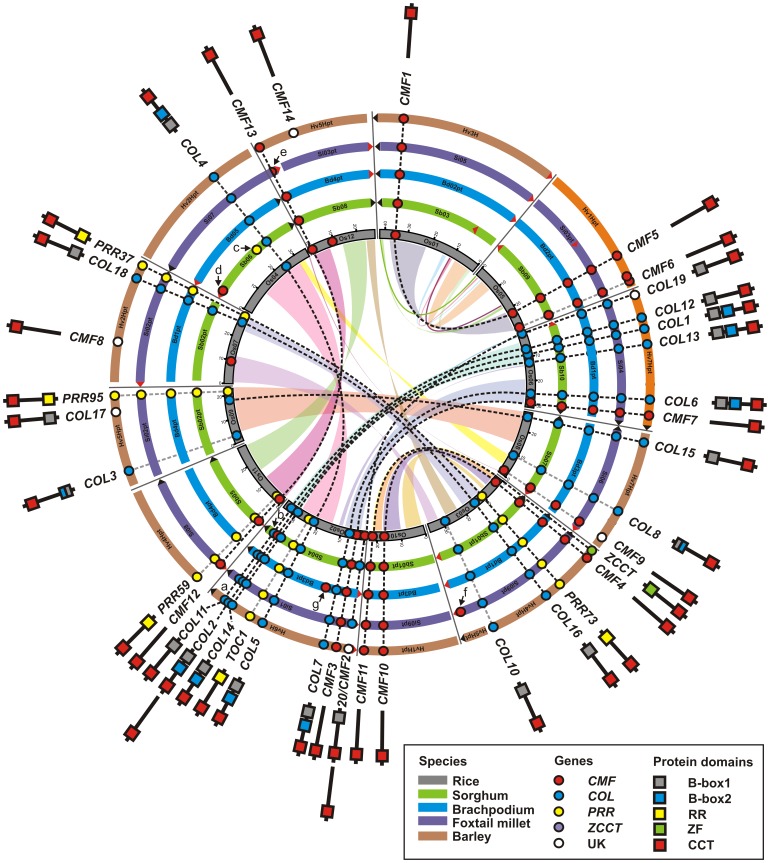
Location of Poaceae *CMF, COL, PRR* and *ZCCT* genes within the framework of Poaceae inter- and intra-specific colinearity. Rice chromosomal regions duplicated during the ancestral WGD are linked by coloured bands. Paralogous gene pairs arising from WGD are connected by black dashed lines, while colinear genes lacking paralogues are connected by grey dashed lines. As instances where full-length barley orthologues were not identified in the draft genomic sequence and fl-CDNAs does not mean they are absent in barley, their predicted positions are indicated tentatively here as unknown (UK) genes. Protein domain configurations are indicated. Localised segmental chromosomal inversions are not shown. ^a^ Poaceae orthologues for *HvCO2* orthologous are present in all genomes except rice [19]. ^b^ *SbCMF16*. ^c^ *SbPRR37*. ^d^ *SbCMF8*. ^e^ Si011862m, truncated CCT motif. ^f^ *SiCMF8*. ^g^ *BdCMF15*.

Comparative mapping allows the genomes of rice, sorghum, brachypodium, foxtail millet and barley to be aligned, and blocks of conserved synteny to be established [49], [51]–[54]. Initially, integration of barley *CMF, COL, PRR* and *ZCCT* genetic map locations into the consensus map (Text S1,S3) allowed investigation of their chromosomal locations within the framework of barley-rice colinearity (Figure 3). All twenty of the mapped barley genes were located within broader regions of established inter-specific colinearity (Text S4). Integration of the ten previously mapped *CMF, COL, PRR* and *ZCCT* genes allowed subsequent comparative analysis all barley gene families to be conducted across five Poaceae species. With the exception of genes present in just one species (*OsCMF14, BdCMF15, OsQ*), colinear cross-species genomic locations of all remaining *CMF, COL* and *PRR* genes was established (Figure 4). Patterns of macro-colinearity were confirmed by investigation of microcolinearity around *CMF, COL* and *PRR* genes in the four sequenced grass genomes (Table S6).

To investigate the gene evolution in the context of intra-specific genome duplication that arose due to WGD in the ancestral grass genome [35]–[36], [49], [52]–[54], the positions of rice *CMF, COL* and *PRR* genes relative to WGD blocks was analysed (Figure 4). The presence of corresponding regions of segmental duplication in each of the sequenced grass species was verified at the micro-synteny level by BLASTn analysis of gene content surrounding all CCT domain genes (Table S6). Combined analysis of inter- and intra-specific colinearity allowed identification of paralogous gene pairs that arose due to ancestral WGD. Of the five *PRR* genes found in each Poaceae species, *PRR37* and *PRR73* appear to have originated from a common ancestor prior to WGD. While the remaining three *PRR* genes originate from regions involved in the WGD, paralogous genes have been lost in all Poaceae species investigated. Furthermore, no *CMF* genes (which could have evolved from *PRR* genes by truncation of the pseudo-receiver domain at the N-terminus) are located in within these genomic locations. Of the seventeen rice *COL* genes and their Poaceae orthologues, the majority form paralogous gene pairs arising from the ancestral WGD (*OsC-OsD*, *OsE*-*OsF*, *OsK-OsL*, *OsM-OsN*, *OsO-OsQ*, *OsP-OsR*). With one exception (due to the absence of *OsQ* orthologues), these paralogous pairs are conserved in all Poaceae species investigated. Of the remaining five *OsCOL* genes, two are located within duplicated genomic regions. *OsA* (chromosome Os06) resides within a chromosomal region displaying intra-specific colinearity with a region of chromosome Os02. *OsA* orthologues are present in all Poaceae species, and while no *OsA* paralogue is present on rice chromosome Os02, brachypodium, sorghum, foxtail millet and barley all retain a paralogue at colinear positions within their genomes. The second *COL* gene located with a region of WGD is *OsS* and its Poaceae orthologues (*COL19*). Although *COL* genes are absent at the corresponding region of chromosomal duplication, *CMF2* genes are present in all species investigated, indicating that Poaceae *OsS* and *CMF2* orthologues are related by evolutionary descent. The remaining Poaceae *COL* genes orthologous to *OsG* and *OsJ* are not located within duplicated chromosomal regions. Of the fourteen members of the rice *CMF* gene family, *OsCMF2* has no orthologous Poaceae *CMF g*enes, as while in rice it appears to have lost the N-terminus B-box domain, this motif is retained in colinear *COL* genes in all other grasses investigated here. All remaining *OsCMF* genes have orthologues located at colinear chromosomal positions in ≥2 grass species. Four *CMF* gene pairs appear to have evolved as a result of the ancestral WGD (*CMF1–5, CMF3–7, CMF4–10, CMF12–13*). In addition, comparative analysis indicates that Poaceae *CMF11* and Triticeae *ZCCT* genes form a paralogous pair originating by ancestral WGD, confirming previous reports in rice/brachypodium/barley [33]. The five remaining grass *CMF* genes (*CMF6, CMF8, CMF9, CMF11, CMF14*) are located in non-duplicated genomic regions.

## Discussion

### Evolution of CCT domain gene families

The role of CCT domain genes in the control of flowering is well documented. However, the recent identification of Poaceae *CO*-like flowering time genes which lack B-boxes [26], [34], and the delimitation of an extended family of related plant *CMF* genes in this study, raises questions concerning their evolutionary origin: are they species-specific independent truncations/mutations of existing *COL* genes, or do they represent a more ancient gene lineage? Here, combined phylogenetic, molecular and comparative analysis identifies predominantly distinct *CMF, COL* and *PRR* gene families in arabidopsis and the Poaceae, showing they predate the monocot/dicot divergence ∼200 million years ago (mya). *COL* [5] and *PRR* [55] genes are present in the moss *Physcomitrella patens*, with BLAST searches showing *CMF* genes to be absent. Mosses within the streptophyte lineage such as the ancestor of *P. patens* separated from the cormophytes ∼500 mya, with the cormophyte lineage (to which the ancestor of the spikemoss *Selaginella moellendorffii* belongs) diverging from seed plants ∼400 mya [56]. Analysis of the *S. moellendorffii* genome (release v1.0) finds both *COL* and *CMF* genes to be present, with ≥1 *CMF* gene(s) within each of the *CMF* clades identified in this study (data not shown). Thus, parsimonious analyses indicate the *CMF* family may have evolved from *COL* genes after the divergence of the cormophyte and streptophyte lineages, around 500−400 mya. Characteristic regions of sequence conservation outside the identified protein domains within and between gene families indicate a hierarchy of evolutionary relatedness resulting from genetic mutation following processes such as WGD, inter-species hybridization events and localized gene duplication or deletion over their evolutionary history. Inter- and intra-species comparative analysis shows that the *CMF, COL* and *PRR* gene families consist largely of arrays of paralogous gene-pairs located at colinear genomic locations across the Poaceae that evolved from common ancestors prior to WGD in the ancestral grass genome. This hypothesis is supported by clustering of Poaceae paralogues within each of the five phylogenetic clades, as well as regions of conservation throughout their proteins. Additionally, phylogenetic and protein/gene structure analyses indicates evolutionary relatedness between groups of *COL* and *CMF* genes prior to monocot/dicot divergence. Within Clade 2, the *CMF* and *COL* Group II sub-branches are phylogeneticly linked, indicating Clade 2 *CMF* genes evolved from Group II *COL* genes, between 400−200 mya. The remaining *CMF* genes are distributed between phylogenetic Clades 4 and 1. While the former consists exclusively of arabidopsis and Poaceae *CMF* genes with a conserved CCT domain intron, the latter contains sub-branches possessing *COL, CMF* and *ZCCT* genes (discussed subsequently). While the majority of *CMF* genes predate monocot/dicot divergence, a minority appear to have evolved more recently (*AtCMF2, AtCMF5, AtCMF7, OsCMF2, SbCMF16*). Such genes cluster unambiguously within *COL* phylogenetic clades, with comparative mapping in the Poaceae indicating they mutated from *COL* genes after separation of the lineages investigated.

All gene families investigated possess a CCT domain in their predicted proteins, with greater amino acid conservation towards the C-terminus. CCT domains possess regions of amino acid conservation with HEME ACTIVATOR PROTEIN2 (HAP2), a component of the HA2/HAP3/HAP5 protein complex that binds to promoter CCAAT DNA motifs in eukaryotic genes, and modifies their transcription [57]. Indeed, arabidopsis *CO* and *COL15* have been shown to interact with several HAP proteins *in vitro* and *in vivo* [57]–[58]. Of the five highly conserved CCT domain residues identified (Tyr^23^-Arg^26^-Ala^30^-Arg^35^-Gly^38^), all but Try^23^ are located in the NF-YA2 sub-domain of HAP2, though to mediate the interaction of HAP2 with CCAAT DNA sequences [59]. These highly conserved CCT/HAP2 residues are likely sites for point mutations resulting in altered function in CMF, COL and PRR proteins. Indeed, independent mutations at three of these conserved residues have been shown to result in novel alleles in several plant species [11], [18], [26], [28], [60] (Figure 1b), Recently, the CCT domain of arabidopsis CO has been shown to mediate protein-protein interaction with the E3 ubiquitin ligases COP1 and HOS1 [60]–[62], both of which regulate CO stability. Taking such data into account, *CMF* gene families are likely to encode transcription factors that modify floral gene expression through DNA-binding or DNA-binding complexes, mediated by the CCT motif.

### Evolution of Poaceae Clade 1 CCT domain genes

Clade 1 contains *CMF, COL* and *ZCCT* genes, distributed between two sub-branches. The first contains *COL* Group I genes from arabidopsis and the grasses, while the second contains *CMF* and *ZCCT* genes, unique to the Poaceae. Common ancestry was demonstrated for four Clade 1 paralogous gene-pairs, which evolved as a result of ancestral cereal WGD (*COL* genes orthologous to *OsA-OsU*, *OsC-OsD, OsE-OsF* and *CMF11-ZCCT*). Interestingly, Clade 1 contains the greatest number of protein domain configurations, including proteins encoded *COL* genes with two B-boxes, or chimeric B-box1/B-box2 domains, as well as *CMF* and *ZCCT* genes. The trend of degradation or complete loss of B-box domains within Clade 1 appears to have resulted in the evolution of CCT domain genes lacking B-boxes within the Poaceae-specific *CMF/ZCCT* sub-branch. The absence of arabidopsis genes within this sub-branch, and the clear grouping (and conserved intron structure) of arabidopsis *COL* genes within the *COL* sub-branch, indicates the *CMF/ZCCT* branch evolved from common ancestors of the *COL* Group I genes after the monocot/dicot divergence. Two examples of Poaceae-specific B-box degradation are evident. In the case of Poaceae *OsB* and *OsG* orthologues in the *COL* sub-branch, the two B-boxes found in all other COL Group I proteins have been fused into one chimeric domain. The presence of chimeric B-boxes in orthologous Poaceae genes shows that truncation occurred prior to the divergence of the grasses, around 60 mya [53]. It has been suggested that *COL* gene evolution is leading to proteins encoding just one B-box domain [13]. The presence of tandemly arrayed B-box domains containing the short stretches of sequence conservation necessary for non-homologous end joining (NHEJ) following double-stranded DNA breaks [63], provides a possible mechanism for B-box contraction. Such flanking motifs have previously been observed in plants surrounding NHEJ deletions within transposable elements [64] and similar deletions within plant genes are responsible for the creation of novel phenotypic variation [49], [65]. As chimeric or degraded B-box domains are absent in arabidopsis COL proteins [13], [26], [29], it appears that the evolutionary process of B-box elimination is prominent in Poaceae Clade 1. Indeed, Clade 1 members illustrate the mechanism by which *CMF* genes may have evolved from *COL* genes after the divergence of the Bryophyta and flowering plants, by creation of non-functional chimeric B-boxes and their subsequent degradation/mutation, as evidenced by the proteins encoded by the paralogous *ZCCT-CMF11* gene pair. The significance of this evolutionary trend in Clade 1 is highlighted by the occurrence of B-box degradation/elimination in two of the three known Poaceae CCT domain flowering time genes, affecting floral response to vernalization in cereals (*ZCCT1* orthologues) and to LD photoperiods in rice (*Ghd7/OsI*). While *Ghd7* orthologues (Poaceae *CMF8* genes) are present in all three tropical SD species investigated, they are not found in either of the two temperate LD cereals investigated (barley and brachypodium). Deletions/mutations causing truncation of Ghd7, and the resulting lack of floral delay under LDs, is one of the key variants that allowed rice to be grown in temperate LD climates [34]. The ancestral cereal is hypothesised to have been a SD plant of tropical environment [1]. Thus, absence of *Ghd7* orthologues in temperate cereals could be explained by their mutation/deletion after the split of temperate and tropical cereal lineages. The resulting removal of LD floral delay would allow growth and reproduction in the warm LD conditions of temperate summers.

It has been suggested that COL proteins act by replacing HAP2 in the HAP2/HAP3/HAP5 transcriptional complex, thus affecting binding to promoter CCAAT motifs of target genes [25], [57]. If the CCT domains of CMF proteins retain similar function, the absence of B-box domains (which mediate protein-protein interactions) could further alter the binding, composition and function of such protein complexes, and their resulting transcriptional modulation. We note that copy number variation for additional Poaceae CCT domain genes has also been critical in the domestication and spread of cereal cultivation, including the *ZCCT* genes at cereal vernalization loci [29]-[30] and *TaPRR* at the hexaploid wheat *PPD-B1* photoperiod locus [21]. Accordingly, the occurrence of species-specific genes (*OsB, OsQ, OsCMF13, BdCMF14*), as well as gene absences (rice *BdCO2*, brachypodium *CMF8* and Poaceae *OsCMF14* orthologues), translocations (*SbCMF8, SiCMF8, SbPRR37*) and duplications (*HvCMF6a/*b), represent genes of potential significance for Poaceae floral control.

### Analysis of CCT domain genes relative to flowering time loci

Three flowering time QTL have been identified in the OWB mapping population used here. The first maps across the *VRN-H2* locus [66], encoded by the *ZCCT-Ha, -Hb, -Hc* genes [29]. Additional QTL have been mapped towards the telomeric region of chromosome 1HL [63]–[64], and between the *Nud* and *Lks2* loci on chromosome 7H [67]. The 1H QTL peak is associated with markers ABG387A and 11_20840, orthologous to Os05g51530 and Os05g51450, respectively [66]–[67]. We find *HvCMF6a* and *HvCMF6b* (orthologous to Os05g51630) to cosegregate with ABG387A and 11_20840 on the long arm of chromosome 1H. Flowering time QTL have been previously mapped in this region in other barley populations [68]–[71], as has the early flowering mutant *early maturity 8* (*eam8*) [67]. Furthermore, QTL for related morphological traits including final leaf number and grain yield are also found at this location (154–158 cM, between markers 11_11509 and 11_20840) [66]. Interestingly, a flowering time QTL is located at a colinear position on the long arm of chromosome 1D in hexaploid wheat [72], based on comparative marker *XBJ544902*. This wheat marker, and barley SNP 11_20840 (which cosegregates with *HvCMF6a/HvCMF6b* and the flowering time QTL in barley), originate from orthologous genes. Environmentally robust flowering time QTL towards the telomeric region of chromosome 1BL and 1DL have been identified in three additional hexaploid wheat mapping populations, leading to the classification of these genomic regions as flowering time meta-QTL, putatively controlled by homeoalleles [72]. Based on known flowering time genes in model species, no additional candidate genes were identified in this region of rice chromosome Os05 (29.00–29.95 Mbp). However, *eam8* has recently been found to encode a homologue of the arabidopsis circadian clock regulator *EARLY FLOWERING 3* (*ELF3*), absent in the colinear region of rice [73]. Nevertheless, it is unclear whether the naturally occurring flowering time QTL in barley and wheat correspond to *ELF3* homologues, with genetic mapping suggesting *eam8* is distal to the barley QTL. Although partial sequencing of *HvCMF6a* did not identify any non-synonymous mutations, *HvCMF6b* was mapped as a presence/absence polymorphism, indicating that deletions, or mutation within primer binding-sites, could result in an *HvCMF6b* allele of altered function in the OWB-R parental line. Further investigation is needed to evaluate the candidacy of *CMF6* and *ELF3* genes for the 1H, 1BL and 1DL meta-QTL. Although *HvCMF7* was mapped to chromosome 7H, it is ∼5 cM distal to *Lks2*, indicating it is an unlikely candidate for the 7H flowering time QTL. However, we note that *HvCO6* (orthologous to Os06g44450) has been mapped to this region in barley [13]. Comparative analysis predicts *HvCO6* to map between *HvCKX10* (Os06g37500) [44] and *HvCMF7* (Os06g48610) in barley, indicating it is a candidate gene for the 7H QTL. Future genetic mapping of *HvCO6* in the OWB population would help clarify its map position relative to *Nud, Lks2* and the 7H flowering time QTL.

## Supporting Information

Figure S1 **Intron (line)/exon (box) structure of rice** ***COL, PRR*** **and** ***CMF*** **genes.** Coding regions encoding protein domains are indicated: CCT (green), B-box1 (blue), B-box2 (red), PRR (orange). Non-significant B-box1 protein domains are indicated in light blue. Synonyms: *OsCMF8* = *OsI* [13] and *Ghd7* [34]. *OsCMF11* = *OsH* [13]. *OsA* = *Hd1* [24] and *OsCO1*. *OsB = OsCO3* [74].
(TIF)

Figure S2 **Alignment of Poaceae and arabidopsis CMF and ZCCT proteins.** ZCCT proteins from *T. monococcum* and *H. vulgare* are included in the alignment. HvCMF1 and HvCMF3 are partial at the N-terminus end. AtCMF2, AtCMF5 and AtCMF7 are excluded.
(PDF)

Figure S3 **Protein alignment of Poaceae COL Group I proteins.** Intron positions are indicated by triangles and boxed. Positions of protein domains are indicated. Intron positions for HvCO1 – HvCO5 were determined from the sequences submitted by [13]. Intron positions for HvCO6, HvCO7 and HvCO8 were determined by gene prediction analysis of cv Morex RBCA contigs 6788, 2171376 and 143637, respectively.
(TIF)

Figure S4 **Protein alignment of COL Group II proteins.** Intron positions are indicated by triangles and boxed. Positions of protein domains are indicated. AtCO8 is excluded due to low sequence conservation with other Group II proteins.
(TIF)

Table S1 **Arabidopsis** ***CMF*** **genes.** Chromosome (chr), amino acids (aa). Synonyms in brackets.
(DOCX)

Table S2 **Poaceae** ***COL*** **genes, and their homologues identified in the sequenced genomes of brachypodium, sorghum and foxtail millet.** *HvCO1 -HvCO8* are previously identified by [13]. Note, *HvCO9* is re-named here as *HvCMF11*, due to lack of B-boxes. Alternative rice *COL* nomenclature shown in parentheses. Note, *HvCO3* is orthologous to *OsB* – no *OsB* orthologues are found in brachypodium, sorghum or foxtail millet. B1 (B-box1), B2 (B-box2), B1/2 (B-box1/B-box2 chimeric domain), CCT (CONSTANS, CO-LIKE, TOC1). ^a^ relative to closest rice homologue. FGENESH reanalysis of: ^b^ scaffold_4, 12553835–12558834 bp; ^c^ scaffold_1, 30543240–30545239 bp; ^d^ scaffold_4:164743–169742 bp; ^e^ scaffold_6:33982724–33992723. ^f^ FGENESH reanalysis of genomic region around annotated gene.
(DOCX)

Table S3 **Poaceae** ***PRR*** **genes, and their homologues identified in the sequenced genomes of brachypodium, sorghum and foxtail millet.** ^a^ relative to closest rice homologue. ^b^ Details according to Sbi1.4 gene set from the Sb1 assembly. Comparison with the allele from genotype ATx623 suggests that this allele corresponds to the *Sbprr37-2* allele, which is predicted to possess a different intron/exon structure [20]. ^c^ No gene model (GM) predicted in the sorghum annotation. Analysis of the sorghum genomic region homologous to Os09g36220 (chromosome Sb2, 65858746–65868745 bp) indicated that a sequencing gap has likely resulted in failure of an appropriate gene model to be formed. Manual inspection shows of the eight exons present in Os09g36220, exon 6 and the 5′ end of exon 7 are predicted to lie within the sorghum sequencing gap. RR (response regulator), CCT (CONSTANS, CO-LIKE, TOC1).
(DOCX)

Table S4 **Poaceae** ***CMF*** **genes, and their homologues identified in the sequenced genomes of brachypodium, sorghum and foxtail millet.** Full length barley *CMF* genes identified in the barley transcriptome/draft genome assembly are also shown. c_ (contig_), N/A (not applicable), B1 (B-box1), B2 (B-box2), B1/2 (B-box1-B-box2 chimeric domain), CCT (CONSTANS, CO-LIKE, TOC1). ^a^ relative to closest rice homologue. ^b^ FGENESH re-analysis of gene model. ^c^ FGENESH gene model. Note: e-value similarity with closest rice homologue is below the arbitrary experimental significance threshold. ^P^ truncated at 5′ end. ^d^ Although genes shown in brackets are most similar to *OsCMF* genes, they possess b-box domains and so belong to the *COL* gene family. Their details are described within the *COL* analyses (Table S2). ^e^ Si011862m, truncated CCT motif.
(DOCX)

Table S5 **Poaceae** ***ZCCT*** **genes investigated in this study.** ^a^ E-values are relative to *ZCCT1* CDS. ^b^ ZF = zinc-finger domain. c partial cDNA sequence only available. N/A = not applicable.
(DOCX)

Table S6 **Analysis of microsynteny.** Rice CDS were used for BLASTn queries to identify genes with significant (≤1.0e-15) homology in the genomes of rice, brachypodium, sorghum and foxtail millet. Where WGD-derived colinear genomic regions are present, micro-colinearity with rice is investigated in both regions. Transposable elements are highlighted in grey. HP = hypothetical protein, CHP = conserved hypothetical protein, EP = expressed protein. N/A = not applicable. * = region of homology is located upstream of gene model. (A) Regions involved in the ancestral WGD. (B) Regions not involved in the ancestral WGD.
(XLSX)

Text S1 **Positioning of previously mapped** ***CMF, COL*** **and** ***PRR*** **genes within the barley consensus genetic map.**
(DOCX)

Text S2 **Genetic mapping of barley genes.**
(DOCX)

Text S3 **Integration of genes genetically mapped in the OWB population into the barley consensus genetic map.**
(DOCX)

Text S4 **Comparative barley-rice analysis of mapped** ***HvCMF, HvCOL*** **and** ***HvPRR*** **genes.**
(DOCX)
