# Supplementary figures and images for: Genome Dynamics Explain the Evolution of Flowering Time CCT Domain Gene Families in the Poaceae
Source: PLoS One. 2012 Sep 24;7(9):e45307. doi: 10.1371/journal.pone.0045307 (PMC3454399; doi:10.1371/journal.pone.0045307)

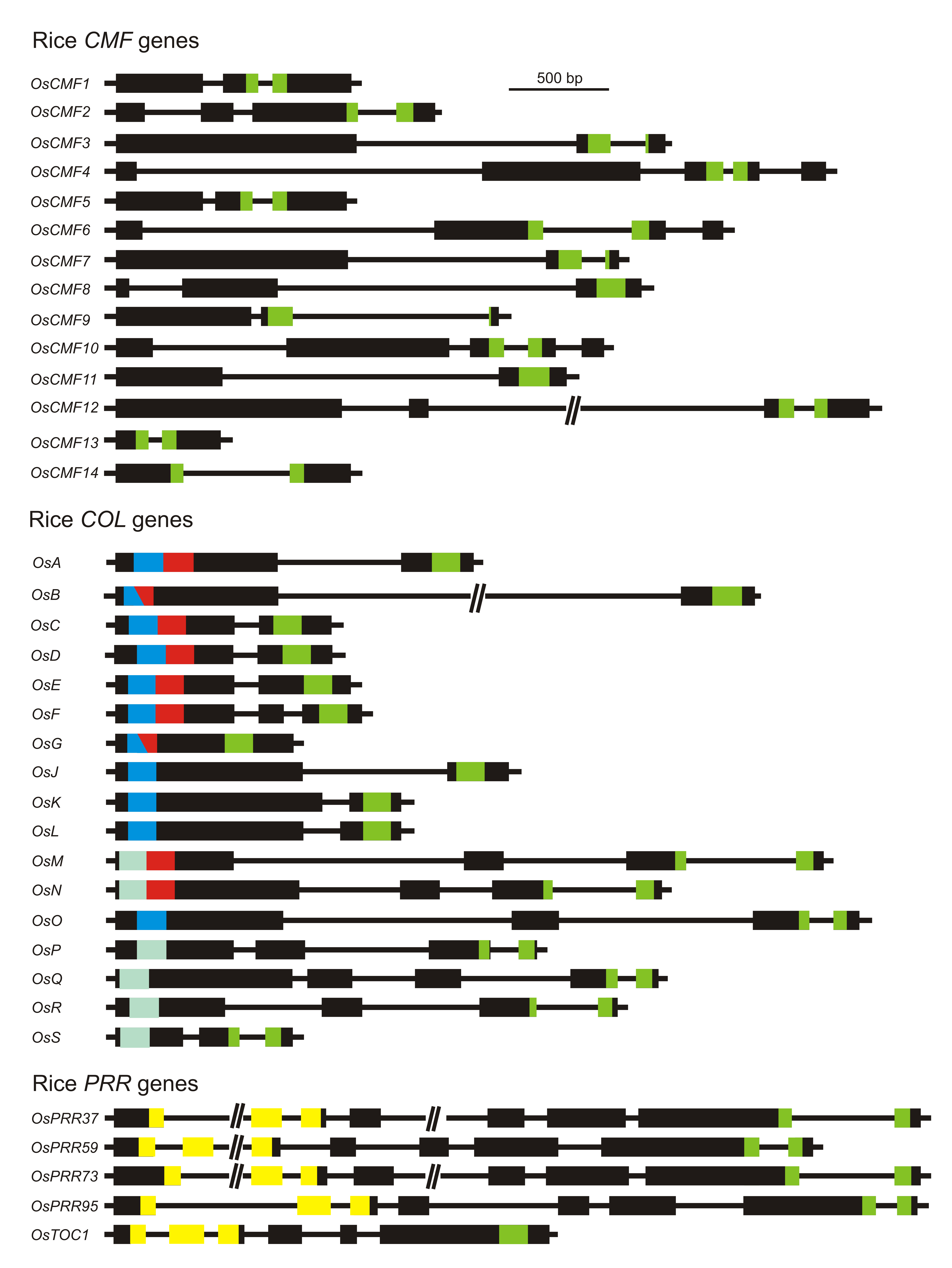

Supplement: Figure S1 — Intron (line)/exon (box) structure of rice COL, PRR and CMF genes. Coding regions encoding protein domains are indicated: CCT (green), B-box1 (blue), B-box2 (red), PRR (orange). Non-significant B-box1 protein domains are indicated in light blue. Synonyms: OsCMF8 = OsI [13] and Ghd7 [34]. OsCMF11 = OsH [13]. OsA = Hd1 [24] and OsCO1. OsB = OsCO3 [74]. (TIF) [file pone.0045307.s001.tif]

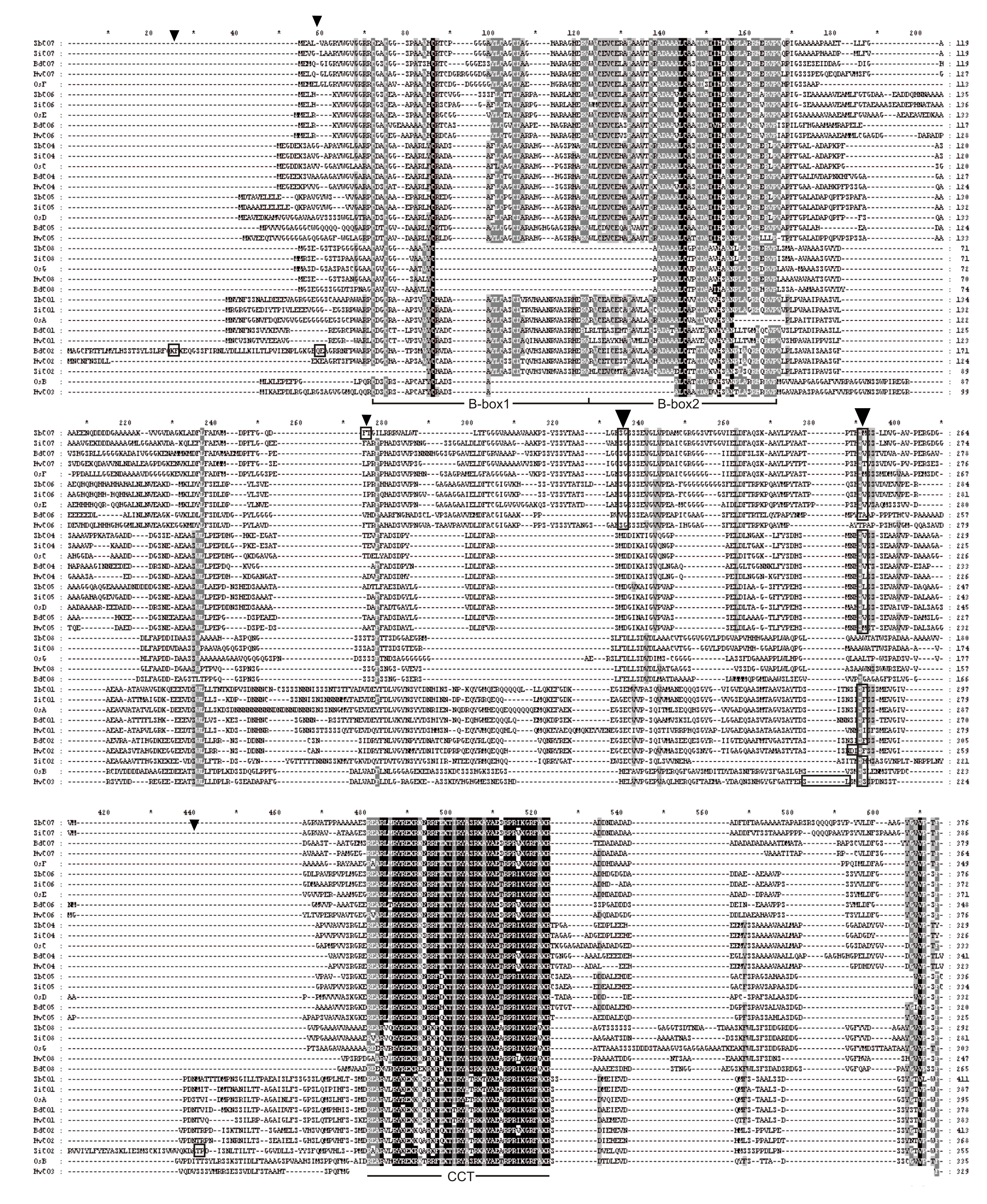

Supplement: Figure S3 — Protein alignment of Poaceae COL Group I proteins. Intron positions are indicated by triangles and boxed. Positions of protein domains are indicated. Intron positions for HvCO1 – HvCO5 were determined from the sequences submitted by [13]. Intron positions for HvCO6, HvCO7 and HvCO8 were determined by gene prediction analysis of cv Morex RBCA contigs 6788, 2171376 and 143637, respectively. (TIF) [file pone.0045307.s003.tif]

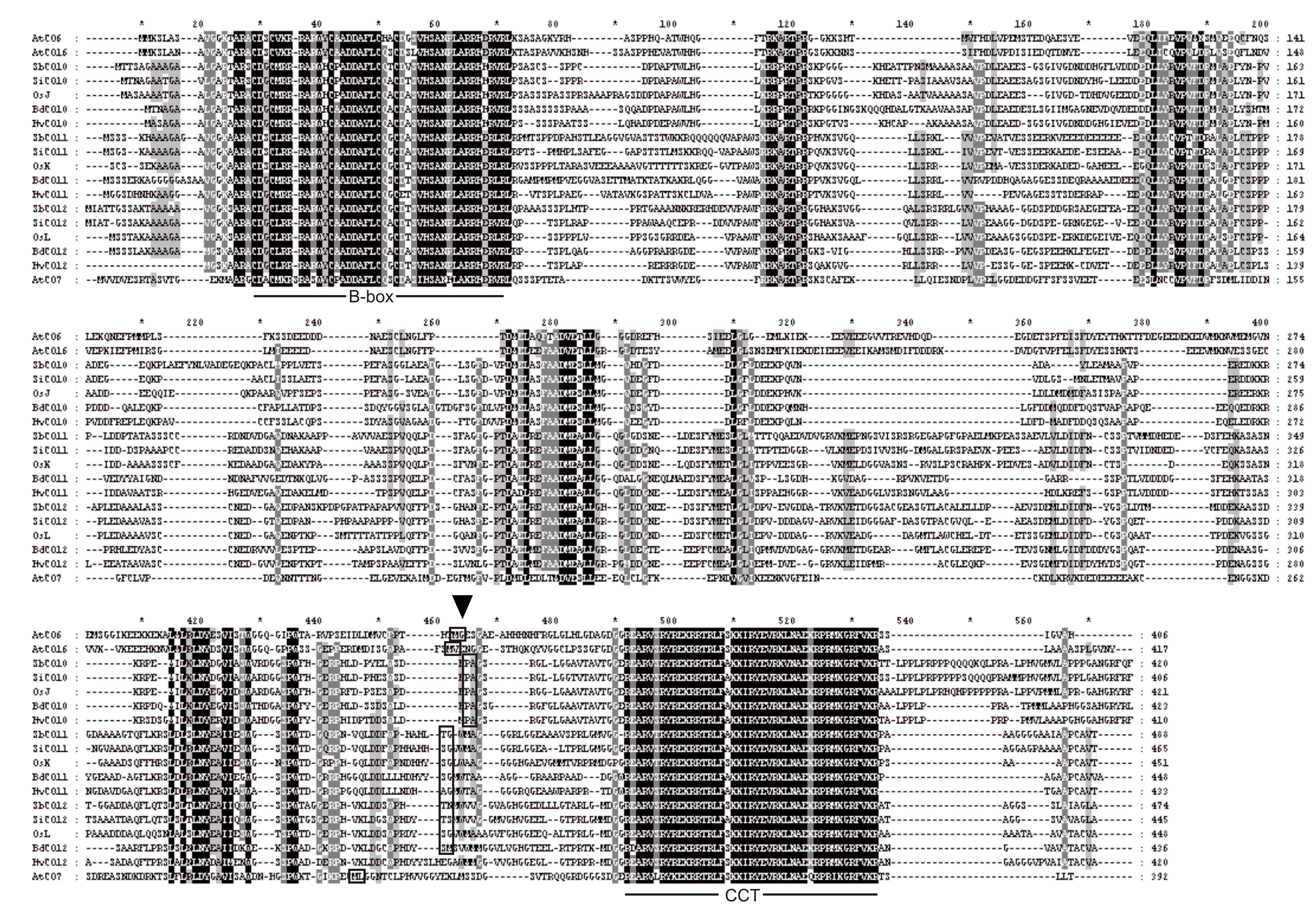

Supplement: Figure S4 — Protein alignment of COL Group II proteins. Intron positions are indicated by triangles and boxed. Positions of protein domains are indicated. AtCO8 is excluded due to low sequence conservation with other Group II proteins. (TIF) [file pone.0045307.s004.tif]
