# Supplementary material for: Genome Dynamics Explain the Evolution of Flowering Time CCT Domain Gene Families in the Poaceae
Source: PLoS One. 2012 Sep 24;7(9):e45307. doi: 10.1371/journal.pone.0045307 (PMC3454399; doi:10.1371/journal.pone.0045307)

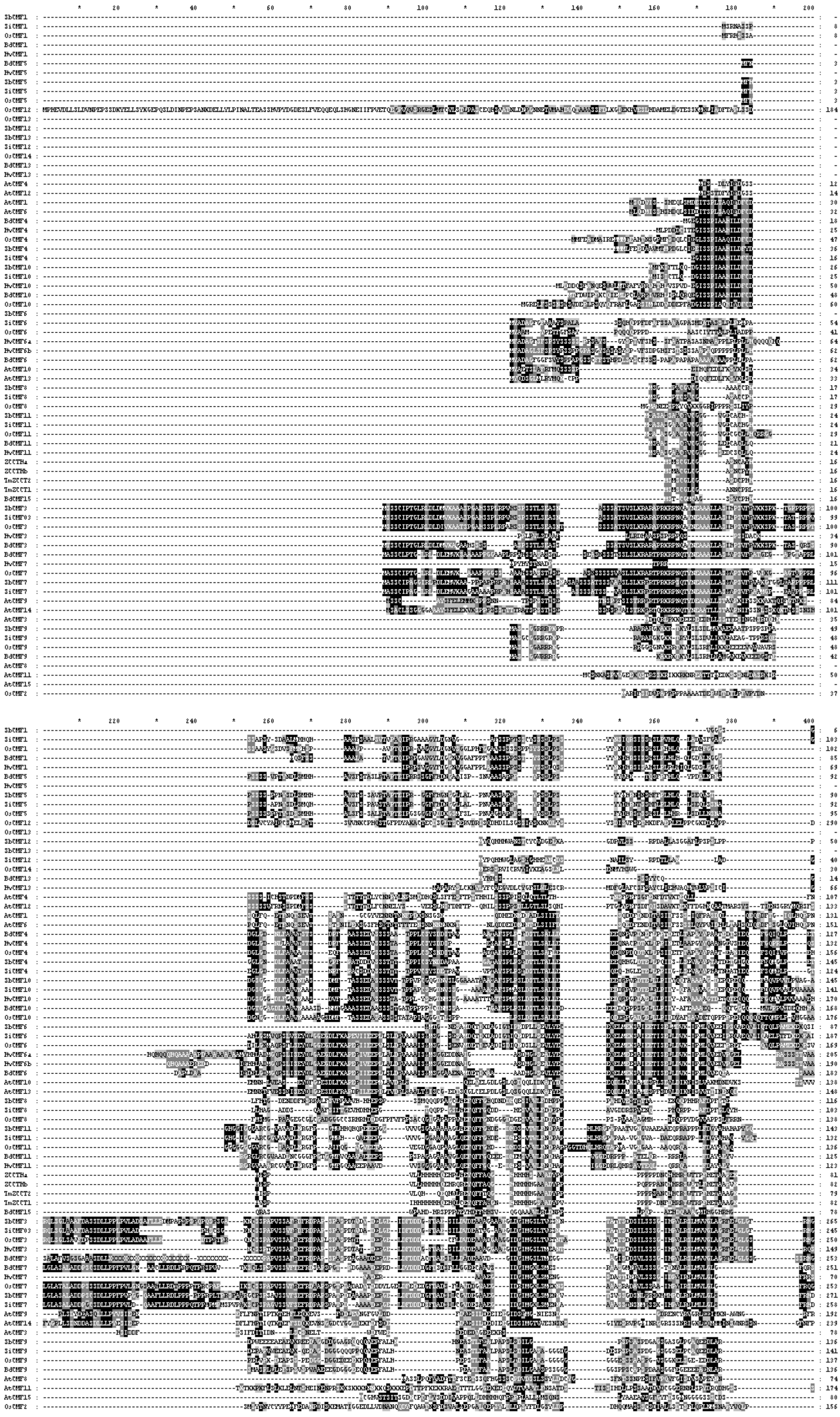

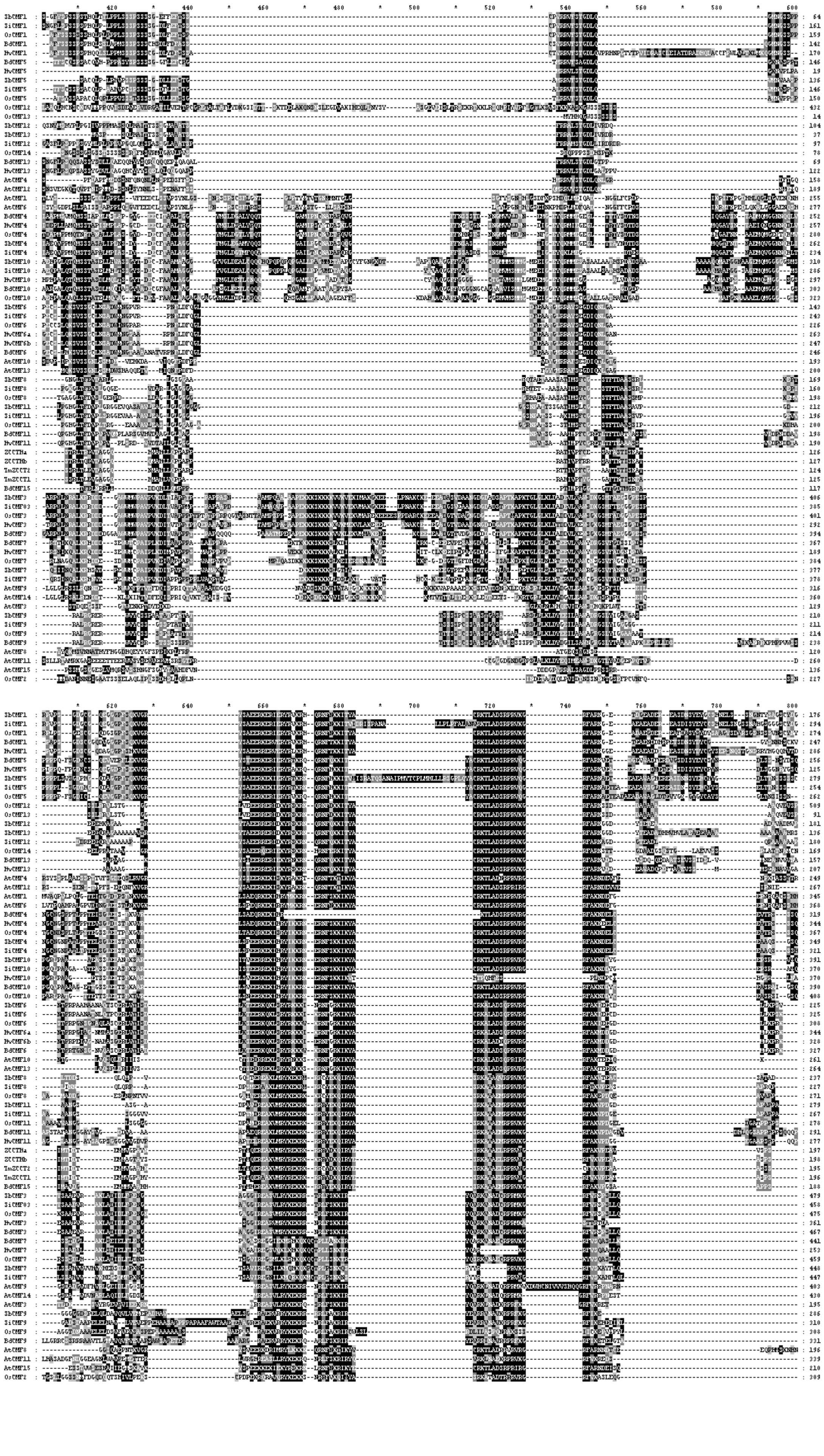

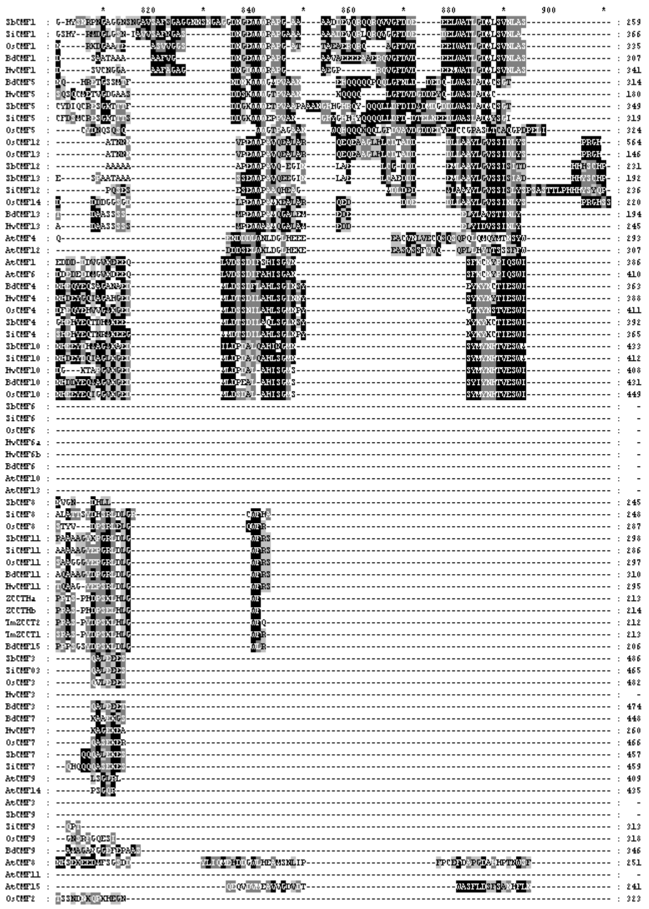

Supplement: Figure S2 — Alignment of Poaceae and arabidopsis CMF and ZCCT proteins. ZCCT proteins from T. monococcum and H. vulgare are included in the alignment. HvCMF1 and HvCMF3 are partial at the N-terminus end. AtCMF2, AtCMF5 and AtCMF7 are excluded. (PDF) [file pone.0045307.s002.pdf]
