# Supplementary material for: Genome Dynamics Explain the Evolution of Flowering Time CCT Domain Gene Families in the Poaceae
Source: PLoS One. 2012 Sep 24;7(9):e45307. doi: 10.1371/journal.pone.0045307 (PMC3454399; doi:10.1371/journal.pone.0045307)
Supplement: Table S1 — Arabidopsis CMF genes. Chromosome (chr), amino acids (aa). Synonyms in brackets. (DOCX) [file pone.0045307.s005.docx]

| **Gene** | **Chr (Mbp)** | **Strand** | **Gene model** | **Genomic (bp)** | **cDNA**  **(bp)** | **Exons** | **Protein (aa)** |
| --- | --- | --- | --- | --- | --- | --- | --- |
|  |  |  |  |  |  |  |  |
| ***A. thaliana*** |  |  |  |  |  |  |  |
| *AtCMF1* | 1 (1.22) | - | *At1g04500* | 2479 | 1161 | 5 | 386 |
| *AtCMF2* | 1 (1.54) | - | *At1g05290* | 1395 | 1110 | 4 | 369 |
| *AtCMF3* | 1 (2.17) | - | *At1g07050* | 807 | 588 | 3 | 195 |
| *AtCMF4* | 1 (23.68) | - | *At1g63820* | 1522 | 882 | 4 | 293 |
| *AtCMF5* | 2 (13.73) | + | *At2g32310* | 1498 | 978 | 7 | 325 |
| *AtCMF6* | 2 (14.13) | + | *At2g33350* | 2720 | 1233 | 5 | 410 |
| *AtCMF7* | 2 (19.17) | - | *At2g46670* | 645 | 552 | 2 | 183 |
| *AtCMF8* (*ASML2*) | 3 (4.02) | + | *At3g12890* | 1055 | 756 | 4 | 251 |
| *AtCMF9* | 4 (13.19) | - | *At4g25990* | 1607 | 1230 | 3 | 409 |
| *AtCMF10* | 4 (13.89) | + | *At4g27900* | 1920 | 786 | 4 | 261 |
| *AtCMF11* | 5 (4.63) | - | *At5g14370* | 1505 | 1020 | 3 | 339 |
| *AtCMF12* | 5 (16.56) | - | *At5g41380* | 1425 | 924 | 4 | 307 |
| *AtCMF13* | 5 (21.67) | + | *At5g53420* | 1787 | 795 | 4 | 264 |
| *AtCMF14* | 5 (23.17) | + | *At5g57180* | 2371 | 1308 | 3 | 435 |
| *AtCMF15* | 5 (24.15) | - | *At5g59990* | 1879 | 726 | 3 | 241 |
|  |  |  |  |  |  |  |  |
|  |  |  |  |  |  |  |  |
|  |  |  |  |  |  |  |  |
