# Supplementary material for: Genome Dynamics Explain the Evolution of Flowering Time CCT Domain Gene Families in the Poaceae
Source: PLoS One. 2012 Sep 24;7(9):e45307. doi: 10.1371/journal.pone.0045307 (PMC3454399; doi:10.1371/journal.pone.0045307)
Supplement: Table S2 — Poaceae COL genes, and their homologues identified in the sequenced genomes of brachypodium, sorghum and foxtail millet. HvCO1 -HvCO8 are previously identified by [13]. Note, HvCO9 is re-named here as HvCMF11, due to lack of B-boxes. Alternative rice COL nomenclature shown in parentheses. Note, HvCO3 is orthologous to OsB – no OsB orthologues are found in brachypodium, sorghum or foxtail millet. B1 (B-box1), B2 (B-box2), B1/2 (B-box1/B-box2 chimeric domain), CCT (CONSTANS, CO-LIKE, TOC1). a relative to closest rice homologue. FGENESH reanalysis of: b scaffold_4, 12553835–12558834 bp; c scaffold_1, 30543240–30545239 bp; d scaffold_4:164743–169742 bp; e scaffold_6:33982724–33992723. f FGENESH reanalysis of genomic region around annotated gene. (DOCX) [file pone.0045307.s006.docx]

|  |  |  |  |  |  |  |  |  |  |  |
| --- | --- | --- | --- | --- | --- | --- | --- | --- | --- | --- |
| **Gene** | **Rice ortho** | **Chr (Mbp)** | **Strand** | **Gene model** | **Genomic (bp)** | **cDNA**  **(bp)** | **Exo-ns** | **Protein (aa)** | **e-value (% identity)^a^** | **Protein domains** |
|  |  |  |  |  |  |  |  |  |  |  |
| ***O. Sativa*** |  |  |  |  |  |  |  |  |  |  |
| *OsA* (*Hd1*) | N/A | 6 (9.34) | + | *Os06g16370* | 1825 | 1188 | 2 | 395 | N/A | B1,B2,CCT |
| *OsB* (*OsCO3*) | N/A | 9 (3.05) | - | *Os09g06464* | 16033 | 1008 | 2 | 335 | N/A | B1/2,CCT |
| *OsC* | N/A | 4 (24.71) | + | *Os04g42020* | 1095 | 1002 | 2 | 333 | N/A | B1,B2,CCT |
| *OsD* | N/A | 2 (23.98) | + | *Os02g39710* | 1080 | 999 | 2 | 332 | N/A | B1,B2,CCT |
| *OsE* | N/A | 6 (26.84) | - | *Os06g44450* | 1203 | 1116 | 2 | 371 | N/A | B1,B2,CCT |
| *OsF* | N/A | 2 (4.32) | + | *Os02g08150* | 1246 | 1050 | 3 | 349 | N/A | B1,B2,CCT |
| *OsG* | N/A | 8 (9.10) | + | *Os08g15050* | 912 | 912 | 1 | 303 | N/A | B1/2,CCT |
| *OsJ* | N/A | 3 (28.68) | + | *Os03g50310* | 1970 | 1266 | 2 | 421 | N/A | B1,CCT |
| *OsK* | N/A | 2 (30.47) | - | *Os02g49880* | 1453 | 1359 | 2 | 452 | N/A | B1,CCT |
| *OsL* | N/A | 6 (8.71) | + | *Os06g15330* | 1446 | 1347 | 2 | 448 | N/A | B1,CCT |
| *OsM* | N/A | 6 (11.07) | - | *Os06g19444* | 3620 | 1227 | 4 | 408 | N/A | B1.B2.CCT |
| *OsN* | N/A | 2 (30.09) | + | *Os02g49230* | 2275 | 1224 | 4 | 407 | N/A | B1,B2,CCT |
| *OsO* | N/A | 8 (26.79) | - | *Os08g42440* | 3749 | 1467 | 4 | 488 | N/A | B1,CCT |
| *OsP* | N/A | 3 (13.15) | + | *Os03g22770* | 2101 | 1212 | 4 | 403 | N/A | B1,CCT |
| *OsQ* | N/A | 9 (19.78) | - | *Os09g33550* | 2732 | 1416 | 4 | 471 | N/A | B1,CCT |
| *OsR* | N/A | 7 (28.19) | - | *Os07g47140* | 2561 | 1143 | 4 | 380 | N/A | B1,CCT |
| *OsS* | N/A | 6 (0.21) | + | *Os06g01340* | 904 | 672 | 3 | 223 | N/A | B1,CCT |
|  |  |  |  |  |  |  |  |  |  |  |
| ***B. distachyon*** |  |  |  |  |  |  |  |  |  |  |
| *BdCO1* | *OsA* | 1 (41.48) | - | *Bradi1g43670* | 2763 | 1137 | 2 | 378 | 3.4e-117 | B1,CCT |
| *BdCO2* | N/A | 3 (56.22) | + | *Bradi3g56260* | 2588 | 1242 | 4 | 413 | N/A | B1,B2,CCT |
| N/A | *OsB* | N/A | N/A | N/A | N/A | N/A | N/A | N/A | N/A | N/A |
| *BdCO4* | *OsC* | 5 (18.05) | + | Bradi5g14600 | 1107 | 1026 | 2 | 341 | 1.1e-179 | B1,B2,CCT |
| *BdCO5* | *OsD* | 3 (49.88) | + | *Bradi3g48450* | 1078 | 1002 | 2 | 333 | 5.2e-133 | B1,B2,CCT |
| *BdCO6* | *OsE* | 1 (26.75) | + | *Bradi1g31280* | 1313 | 1047 | 3 | 348 | 4.8e-77 | B1,B2,CCT |
| *BdCO7* | *OsF* | 3 (4.13) | + | *Bradi3g05800* | 1341 | 1140 | 3 | 379 | 1.7e-101 | B1,B2,CCT |
| *BdCO8* | *OsG* | 3 (17.62) | + | *Bradi3g19010* | 798 | 798 | 1 | 265 | 1.9e-87 | B1/2,CCT |
| *BdCO10* | *OsJ* | 1 (8.36) | + | *Bradi1g11310* | 1387 | 1272 | 2 | 423 | e=0 | B1,CCT |
| *BdCO11* | *OsK* | 3 (56.70) | + | *Bradi3g57000* | 1437 | 1347 | 2 | 448 | 3.9e-136 | B1,CCT |
| *BdCO12* | *OsL* | 1 (41.91) | - | *Bradi1g43990* | 1432 | 1311 | 2 | 436 | 1.5e-179 | B1,CCT |
| *BdCO13* | *OsM* | 1 (40.83) | + | Bradi1g43220 | 4749 | 1206 | 4 | 401 | e=0 | B1,B2,CCT |
| *BdCO14* | *OsN* | 3 (56.35) | - | *Bradi3g56490* | 3260 | 1182 | 4 | 393 | 1.4e-166 | B1,B2,CCT |
| *BdCO15* | *OsO* | 3 (43.42) | - | *Bradi3g41500* | 3649 | 1473 | 4 | 490 | 1.5e-116 | B1,CCT |
| *BdCO16* | *OsP* | 1 (61.65) | - | *Bradi1g62420* | 2047 | 1137 | 4 | 378 | 3.3e-111 | B1,CCT |
| *BdCO17* | *OsQ* | N/A | N/A | N/A | N/A | N/A | N/A | N/A | N/A | N/A |
| *BdCO18* | *OsR* | 1 (14.77) | - | *Bradi1g18410* | 1897 | 1353 | 5 | 450 | 2.0e-31 | B1,CCT |
| *BdCO19* | *OsS* | 1 (50.67) | + | *Bradi1g52360* | 2317 | 1182 | 4 | 393 | 1.8e-16 | B1,CCT |
|  |  |  |  |  |  |  |  |  |  |  |
|  |  |  |  |  |  |  |  |  |  |  |
| ***S. bicolor*** |  |  |  |  |  |  |  |  |  |  |
| *SbCO1* | *OsA* | 10 (12.28) | + | *Sb10g010050* | 1490 | 1236 | 2 | 411 | 2.1e-93 | B1,B2,CCT |
| N/A | *OsB* | N/A | N/A | N/A | N/A | N/A | N/A | N/A | N/A | N/A |
| *SbCO4* | *OsC* | 6 (50.74) | + | *Sb06g021480* | 1104 | 990 | 2 | 329 | e=0 | B1,B2,CCT |
| *SbCO5* | *OsD* | 4 (55.44) | + | *Sb04g025660* | 1141 | 1011 | 2 | 336 | e=0 | B1,B2,CCT |
| *SbCo6* | *OsE* | 10 (55.39) | - | *Sb10g026060* | 1345 | 1131 | 3 | 376 | e=0 | B1,B2,CCT |
| *SbCO7* | *OsF* | 4 (5.07) | + | *Sb04g005250* | 1404 | 1131 | 4 | 376 | 2.4e-117 | B1,B2,CCT |
| *SbCO8* | *OsG* | 7 (14.41) | - | *Sb07g008550* | 879 | 879 | 1 | 292 | 6.7e-130 | B1/2,CCT |
| *SbCO10* | *OsJ* | 1 (9.25) | - | *Sb01g010420* | 1387 | 1263 | 2 | 420 | e=0 | B1,CCT |
| *SbCO11* | *OsK* | 4 (58.99) | + | *Sb04g028920* | 1605 | 1467 | 2 | 488 | e=0 | B1,CCT |
| *SbCO12* | *OsL* | 10 (10.70) | + | *Sb10g009480* | 1518 | 1425 | 2 | 474 | e=0 | B1,CCT |
| *SbCO13* | *OsM* | 10 (14.42) | - | *Sb10g010860* | 2829 | 1221 | 4 | 406 | e=0 | B1,B2,CCT |
| *SbCO14* | *OsN* | 4 (59.57) | - | *Sb04g029480* | 2370 | 1221 | 4 | 406 | 3.4e-154 | B1,B2,CCT |
| *SbCO15* | *OsO* | 7 (61.09) | + | *Sb07g025940* | 3658 | 1473 | 4 | 490 | 2.2e-157 | B1,CCT |
| *SbCO16* | *OsP* | 1 (58.95) | - | *Sb01g035400* | 2534 | 1242 | 2 | 413 | 1.6e-107 | B1,CCT |
| *SbCO17* | *OsQ* | N/A | N/A | N/A | N/A | N/A | N/A | N/A | N/A | N/A |
| *SbCO18* | *OsR* | 2 (75.93) | - | *Sb02g042230* | 2272 | 1242 | 4 | 413 | 5.0e-82 | B1,CCT |
| *SbCO19* | *OsS* | 10 (0.12) | + | *Sb10g000320* | 1161 | 972 | 3 | 323 | 4.0e-17 | B1,CCT |
| *SbCO20* | N/A | 4 (0.68) | - | *Sb04g000820* | 2956 | 1464 | 4 | 487 | N/A | B1,B2,CCT |
|  |  |  |  |  |  |  |  |  |  |  |
|  |  |  |  |  |  |  |  |  |  |  |
| ***S. italica*** |  |  |  |  |  |  |  |  |  |  |
| *SiCO1* | *OsA* | 4 (12.56) | + | *SiFGENESH1*^b^ | 2362 | 1197 | 2 | 398 | 2.7e-114 | B1,B2,CCT |
| *SiCO2* | N/A | 1 (36.91) | - | *Si019803m.g* | 1620 | 1068 | 3 | 355 | N/A | B1,B2,CCT |
| *SiCO3* | *OsB* | N/A | N/A | N/A | N/A | N/A | N/A | N/A | N/A | N/A |
| *SiCO4* | *OsC* | 7 (23.93) | + | *Si010592m.g* | 1102 | 981 | 2 | 326 | 4.3e-155 | B1,B2,CCT |
| *SiCO5* | *OsD* | 1 (30.54) | + | *SiFGENESH2*^c^ | 1147 | 1005 | 2 | 334 | 1.1e-86 | B1,B2,CCT |
| *SiCO6* | *OsE* | 4 (31.04) | + | *Si006690m.g* | 1321 | 1119 | 3 | 372 | 7.1e-153 | B1,B2,CCT |
| *SiCO7* | *OsF* | 1 (6.11) | - | *Si017487m.g* | 1395 | 1161 | 3 | 386 | 1.1e-105 | B1,B2,CCT |
| *SiCO8* | *OsG* | 6 (10.23) | - | *Si014224m.g* | 846 | 846 | 1 | 281 | 2.2e-120 | B1/2,CCT |
| *SiCO10* | *OsJ* | 9 (7.44) | - | *Si035937m.g* | 1335 | 1221 | 2 | 406 | e=0 | B1,CCT |
| *SiCO11* | *OsK* | 1 (37.16) | - | *Si017124m.g* | 1505 | 1398 | 2 | 465 | e=0 | B1,CCT |
| *SiCO12* | *OsL* | 4 (11.41) | + | *Si006432m.g* | 1439 | 1338 | 2 | 445 | e=0 | B1,CCT |
| *SiCO13* | *OsM* | 2 (26.39) | + | *Si030034m.g* | 3466 | 1221 | 4 | 406 | e=0 | B1,B2,CCT |
| *SiCO14* | *OsN* | 1 (36.70) | + | *Si017374m.g* | 2598 | 1224 | 4 | 407 | 8.0e-71 | B1,B2,CCT |
| *SiCO15* | *OsO* | 6 (33.99) | - | *Si014037m.g*^e^ | 3582 | 1716 | 6 | 571 | 1.7e-168 | B1,CCT |
| *SiCO16* | *OsP* | 9 (46.37) | - | *Si034611m.g*^f^ | 5083 | 1726 | 7 | 575 | 3.1e-107 | B1,CCT |
| *SiCO17* | *OsQ* | N/A | N/A | N/A | N/A | N/A | N/A | N/A | N/A | N/A |
| *SiCO18* | *OsR* | 2 (47.69) | - | *Si030140m.g* | 1820 | 1155 | 4 | 384 | 1.6e-34 | B1,CCT |
| *SiCO19* | *OsS* | 4 (0.12) | + | *SiFGENESH3*^d^ | 939 | 840 | 2 | 279 | 1.5e-19 | B1,CCT |
| *SiCO20* | N/A | 1 (0.47) | - | *Si019213m* | 2341 | 1299 | 4 | 432 | N/A | B1,CCT |
|  |  |  |  |  |  |  |  |  |  |  |
|  |  |  |  |  |  |  |  |  |  |  |
| ***H. vulgare*** |  |  |  |  |  |  |  |  |  |  |
| *HvCO10* | *OsJ* | N/A | N/A | AK373259 | N/A | 1230 | N/A | 410 | e=0 | B1,CCT |
| *HvCO11* | *OsK* | N/A | N/A | AK356535 | N/A | 1302 | N/A | 433 | e=0 | B1,CCT |
| *HvCO12* | *OsL* | 7HS | N/A | c_125603 | 1260 | 1260 | 1 | 420 | e=0 | B1,CCT |
| *HvCO13* | *OsM* | 7HS | N/A | c_1012573 | 3270 | 1374 | 5 | 458 | e=0 | B1,B2,CCT |
| *HvCO14* | *OsN* | 6HL | N/A | c_351543 | 2204 | 1146 | 4 | 382 | 1.0e-146 | B1,B2,CCT |
| *HvCO15* | *OsO* | N/A | N/A | AK355971 | N/A | 1461 | N/A | 487 | e=0 | B1,CCT |
| *HvCO16* | *OsP* | N/A | N/A | AK361959 | N/A | 1370 | N/A | 457 | e=0 | B1,CCT |
| *HvCO18* | *OsR* | N/A | N/A | AK370596 | N/A | 1131 | N/A | 377 | 7.0e-96 | B1,CCT |
|  |  |  |  |  |  |  |  |  |  |  |
|  |  |  |  |  |  |  |  |  |  |  |
