# Supplementary material for: Genome Dynamics Explain the Evolution of Flowering Time CCT Domain Gene Families in the Poaceae
Source: PLoS One. 2012 Sep 24;7(9):e45307. doi: 10.1371/journal.pone.0045307 (PMC3454399; doi:10.1371/journal.pone.0045307)
Supplement: Table S3 — Poaceae PRR genes, and their homologues identified in the sequenced genomes of brachypodium, sorghum and foxtail millet. a relative to closest rice homologue. b Details according to Sbi1.4 gene set from the Sb1 assembly. Comparison with the allele from genotype ATx623 suggests that this allele corresponds to the Sbprr37-2 allele, which is predicted to possess a different intron/exon structure [20]. c No gene model (GM) predicted in the sorghum annotation. Analysis of the sorghum genomic region homologous to Os09g36220 (chromosome Sb2, 65858746–65868745 bp) indicated that a sequencing gap has likely resulted in failure of an appropriate gene model to be formed. Manual inspection shows of the eight exons present in Os09g36220, exon 6 and the 5′ end of exon 7 are predicted to lie within the sorghum sequencing gap. RR (response regulator), CCT (CONSTANS, CO-LIKE, TOC1). (DOCX) [file pone.0045307.s007.docx]

|  |  |  |  |  |  |  |  |  |  |
| --- | --- | --- | --- | --- | --- | --- | --- | --- | --- |
| **Gene** | **Chr (Mbp)** | **Strand** | **Gene model** | **Genomic (bp)** | **cDNA**  **(bp)** | **Exo-ns** | **Protein (aa)** | **e-value (% identity)^a^** | **Protein domains** |
|  |  |  |  |  |  |  |  |  |  |
| ***O. Sativa*** |  |  |  |  |  |  |  |  |  |
| *OsPRR37* | 7 (29.62) | + | *Os07g49460* | 11171 | 2229 | 8 | 742 | N/A | RR,CCT |
| *OsPRR59* | 11 (2.71) | + | *Os11g05930* | 4476 | 2100 | 8 | 699 | N/A | RR,CCT |
| *OsPRR73* | 3 (9.77) | - | *Os03g17570* | 7622 | 2304 | 8 | 767 | N/A | RR,CCT |
| *OsPRR95* | 9 (20.89) | - | *Os09g36220* | 4069 | 1872 | 8 | 623 | N/A | RR,CCT |
| *OsTOC1* | 2 (24.57) | + | *Os02g40510* | 2200 | 1557 | 6 | 518 | N/A | RR,CCT |
|  |  |  |  |  |  |  |  |  |  |
| ***B. distachyon*** |  |  |  |  |  |  |  |  |  |
| *BdPRR37* | 1 (13.35) | - | *Bradi1g16490* | 3238 | 1983 | 8 | 660 | 1.6e-105 | RR,CCT |
| *BdPRR59* | 4 (30.22) | + | *Bradi4g24967* | 4894 | 2160 | 8 | 720 | e=0 | RR,CCT |
| *BdPRR73* | 1 (64.80) | + | *Bradi1g65910* | 6208 | 2301 | 8 | 766 | e=0 | RR,CCT |
| *BdPRR95* | 4 (41.35) | - | *Bradi4g36077* | 3696 | 1866 | 8 | 622 | 9.9e-133 | RR,CCT |
| *BdTOC1* | 3 (50.31) | + | *Bradi3g48880* | 2174 | 1566 | 6 | 521 | e=0 | RR,CCT |
|  |  |  |  |  |  |  |  |  |  |
|  |  |  |  |  |  |  |  |  |  |
| ***S. bicolor*** |  |  |  |  |  |  |  |  |  |
| *SbPRR37* (*Ma1*) | 6 (40.28) | + | *Sb06g014570^b^* | 10189 | 1842 | 7 | 613 | 2.5e-165 | RR,CCT |
| *SbPRR59* | 5 (4.26) | + | *Sb05g003660* | 4380 | 2094 | 8 | 697 | 1.8e-179 | RR,CCT |
| *SbPRR73* | 1 (62.31) | + | *Sb01g038820* | 5735 | 2298 | 8 | 765 | e=0 | RR,CCT |
| *SbPRR95* | 2 (65.87) | - | *Partial GM^c^* | >3512 | >1164 | ≥7 | >388 | 4.3e-60 | RR,CCT |
| *SbTOC1* | 4 (55.96) | + | *Sb04g026190* | 2346 | 1575 | 6 | 524 | e=0 | RR,CCT |
|  |  |  |  |  |  |  |  |  |  |
|  |  |  |  |  |  |  |  |  |  |
| ***S. italica*** |  |  |  |  |  |  |  |  |  |
| *SiPRR37* | 2 (49.13) | + | *Si033274m* | 5131 | 1986 | 7 | 661 | 1.1e-96 | RR,CCT |
| *SiPRR59* | 8 (2.91) | + | *Si026170m* | 2846 | 1701 | 6 | 566 | e=0 | CCT |
| *SiPRR73* | 9 (49.51) | + | *Si034368m* | 6775 | 2283 | 8 | 760 | e=0 | RR,CCT |
| *SiPRR95* | 2 (38.25) | - | *Si029202m* | 4154 | 1893 | 8 | 630 | 7.2e-143 | RR,CCT |
| *SiTOC1* | 1 (31.45) | + | *Si016922m* | 2266 | 1557 | 6 | 518 | e=0 | RR,CCT |
|  |  |  |  |  |  |  |  |  |  |
|  |  |  |  |  |  |  |  |  |  |
| ***H. vulgare*** |  |  |  |  |  |  |  |  |  |
| *PPD-H1* | 2H | N/A | *AY943294* | 3128 | 2025 | 8 | 674 | 4.0e-92 | RR,CCT |
| *HvPRR59* | N/A | N/A | *c_159210* | >4607 | >1884 | >7 | >627 | e=0 | RR,CCT |
| *HvPRR73* | N/A | N/A | *c_5383* | 4523 | 2280 | 8 | 759 | 3.0e-180 | RR,CCT |
| *HvPRR95* | N/A | N/A | *c_4678* | 3121 | 2256 | 7 | 604 | 5.0e-101 | RR,CCT |
| *HvTOC1* | N/A | N/A | *c_123808* | 2263 | 1809 | 6 | 602 | e=0 | RR,CCT |
|  |  |  |  |  |  |  |  |  |  |
