# Supplementary material for: Genome Dynamics Explain the Evolution of Flowering Time CCT Domain Gene Families in the Poaceae
Source: PLoS One. 2012 Sep 24;7(9):e45307. doi: 10.1371/journal.pone.0045307 (PMC3454399; doi:10.1371/journal.pone.0045307)
Supplement: Table S4 — Poaceae CMF genes, and their homologues identified in the sequenced genomes of brachypodium, sorghum and foxtail millet. Full length barley CMF genes identified in the barley transcriptome/draft genome assembly are also shown. c_ (contig_), N/A (not applicable), B1 (B-box1), B2 (B-box2), B1/2 (B-box1-B-box2 chimeric domain), CCT (CONSTANS, CO-LIKE, TOC1). a relative to closest rice homologue. b FGENESH re-analysis of gene model. c FGENESH gene model. Note: e-value similarity with closest rice homologue is below the arbitrary experimental significance threshold. P truncated at 5′ end. d Although genes shown in brackets are most similar to OsCMF genes, they possess b-box domains and so belong to the COL gene family. Their details are described within the COL analyses (Table S2). e Si011862m, truncated CCT motif. (DOCX) [file pone.0045307.s008.docx]

|  |  |  |  |  |  |  |  |  |  |
| --- | --- | --- | --- | --- | --- | --- | --- | --- | --- |
| **Gene** | **Chr (Mbp)** | **Strand** | **Gene model** | **Genomic (bp)** | **cDNA**  **(bp)** | **Exo-ns** | **Protein (aa)** | **e-value (% identity)^a^** | **Protein domain** |
|  |  |  |  |  |  |  |  |  |  |
| ***O. Sativa*** |  |  |  |  |  |  |  |  |  |
| *OsCMF1* | 1 (35.83) | - | *Os01g61900* | 1639 | 1008 | 3 | 335 | N/A | CCT |
| *OsCMF2* | 2 (0.55) | - | *Os02g01990* | 1558 | 972 | 4 | 323 | N/A | CCT |
| *OsCMF3* | 2 (2.64) | + | *Os02g05470* | 3695 | 1449 | 3 | 482 | N/A | CCT |
| *OsCMF4* | 3 (2.16) | - | *Os03g04620* | 4030 | 1236 | 5 | 411 | N/A | CCT |
| *OsCMF5* | 5 (22.81) | - | *Os05g38990* | 1576 | 975 | 3 | 324 | N/A | CCT |
| *OsCMF6* | 5 (29.60) | + | *Os05g51690* | 3772 | 927 | 4 | 308 | N/A | CCT |
| *OsCMF7* | 6 (29.41) | + | *Os06g48610* | 4295 | 1401 | 3 | 466 | N/A | CCT |
| *OsCMF8 (OsI,Ghd7)* | 7 (9.15) | - | *Os07g15770* | 2784 | 864 | 3 | 287 | N/A | CCT |
| *OsCMF9* | 8 (1.08) | - | *Os08g02620*^b^ | 2514 | 912 | 3 | 304^$^ | N/A | CCT |
| *OsCMF10* | 10 (17.15) | + | *Os10g32900* | 2936 | 1350 | 5 | 449 | N/A | CCT |
| *OsCMF11 (OsH)* | 10 (22.00) | - | *Os10g41100* | 2821 | 894 | 2 | 297 | N/A | CCT |
| *OsCMF12* | 11 (0.44) | + | *Os11g01074.4* | 5850 | 1695 | 4 | 564 | N/A | CCT |
| *OsCMF13* | 12 (0.04) | + | *Os12g01080* | 533 | 441 | 2 | 146 | N/A | CCT |
| *OsCMF14* | 12 (9.23) | - | *Os12g16160* | 1188 | 663 | 2 | 220 | N/A | CCT |
|  |  |  |  |  |  |  |  |  |  |
| ***B. distachyon*** |  |  |  |  |  |  |  |  |  |
| *BdCMF1* | 2 (53.22) | - | *Bradi2g54260* | 1134 | 924 | 3 | 307 | 1.8e-75 | CCT |
| *BdCMF2* | N/A | N/A | N/A | N/A | N/A | N/A | N/A | N/A | N/A |
| *BdCMF3* | 3 (2.51) | + | *Bradi3g03770* | 2822 | 1425 | 3 | 474 | 1.5e-173 | CCT |
| *BdCMF4* | 1 (72.57) | + | *Bradi1g75760* | 1825 | 1092 | 5 | 363 | e=0 | CCT |
| *BdCMF5* | 2 (20.30) | + | *Bradi2g22800* | 1136 | 945 | 3 | 314 | 2.6e-73 | CCT |
| *BdCMF6* | 2 (12.84) | - | *Bradi2g14220* | 3173 | 984 | 4 | 327 | 1.1e-71 | CCT |
| *BdCMF7* | 1 (29.64) | + | *Bradi1g34060* | 3388 | 1347 | 3 | 448 | 5.3e-179 | CCT |
| *BdCMF8* | N/A | N/A | N/A | N/A | N/A | N/A | N/A | N/A | N/A |
| *BdCMF9* | 3 (12.42) | - | *Bradi3g13960* | 1513 | 1041 | 3 | 346 | 1.1e-71 | CCT |
| *BdCMF10* | 3 (29.60) | + | *Bradi3g28290* | 2119 | 1296 | 6 | 431 | 3.0e-55 | CCT |
| *BdCMF11* | 3 (35.71) | - | *Bradi3g33340* | 1082 | 933 | 2 | 310 | 7.9e-67 | CCT |
| *BdCMF12* | N/A | N/A | N/A | N/A | N/A | N/A | N/A | N/A | N/A |
| *BdCMF13*^b^ | 4 (48.46) | - | *Bradi4g45330* | 602 | 474 | 2 | 157 | N/A | CCT |
| *BdCMF14* | N/A | - | N/A | N/A | N/A | N/A | N/A | N/A | N/A |
| *BdCMF15* | 3 (8.25) | - | *Bradi3g10010* | 1724 | 621 | 2 | 206 | 2.2e-29 | CCT |
|  |  |  |  |  |  |  |  |  |  |
|  |  |  |  |  |  |  |  |  |  |
| ***S. bicolor*** |  |  |  |  |  |  |  |  |  |
| *SbCMF1* | 3 (66.86) | - | *Sb03g039060* | 1339 | 1080 | 3 | 359 | 5.2e-62 | CCT |
| *SbCMF2* | N/A | N/A | (Sb04g000820)^d^ | N/A | N/A | N/A | N/A | N/A | N/A |
| *SbCMF3* | 4 (3.32) | + | *Sb04g003470* | 2678 | 1461 | 3 | 486 | e=0 | CCT |
| *SbCMF4* | 1 (68.00) | - | *Sb01g047650* | 2393 | 999 | 5 | 332 | 9.7e-117 | CCT |
| *SbCMF5* | 9 (52.55) | - | *Sb09g022900* | 1192 | 1050 | 3 | 349 | 9.9e-84 | CCT |
| *SbCMF6* | 9 (59.47) | + | *Sb09g030830* | 1279 | 678 | 3 | 225 | 1.1e-139 | CCT |
| *SbCMF7* | 10 (58.67) | + | *Sb10g028840* | 2904 | 1374 | 4 | 457 | 2.6e-156 | CCT |
| *SbCMF8* | 6 (0.67) | + | *Sb06g000570* | 2643 | 738 | 3 | 245 | 9.4e-33 | CCT |
| *SbCMF9* | 7 (2.19) | - | *Sb07g002040* | 976 | 852 | 2 | 283 | 7.5e-60 | CCT |
| *SbCMF10* | 1 (21.64) | - | *Sb01g019780* | 1793 | 1302 | 5 | 433 | 1.3e-77 | CCT |
| *SbCMF11* | 1 (50.82) | + | *Sb01g029080* | 2728 | 897 | 2 | 298 | 1.6e-80 | CCT |
| *SbCMF12* | 5 (0.06) | + | *Sb05g000230* | 766 | 696 | 2 | 231 | 1.2e-21 | CCT |
| *SbCMF13* | 8 (0.49) | + | *Sb08g000240* | 724 | 579 | 2 | 192 | 3.5e-24 | CCT |
| *SbCMF14* | N/A | N/A | N/A | N/A | N/A | N/A | N/A | N/A | N/A |
| *SbCMF16* (*SbCO2*) | 4 (59.19) | + | *Sb04g029180* | 1504 | 870 | 3 | 289 | N/A | CCT |
|  |  |  |  |  |  |  |  |  |  |
|  |  |  |  |  |  |  |  |  |  |
| ***S. italica*** |  |  |  |  |  |  |  |  |  |
| *SiCMF1* | 5 (40.86) | - | *Si_MODEL1*^c^ | 1259 | 1104 | 3 | 367 | 1.7e-52 | CCT |
| *SiCMF2* | N/A | N/A | (*Si019213m.g*)^d^ | N/A | N/A | N/A | N/A | N/A | N/A |
| *SiCMF3* | 1 (7.89) | - | *Si017125m.g* | 2890 | 1398 | 4 | 465 | e=0 | CCT |
| *SiCMF4* | 9 (56.61) | + | *Si039219m.g* | 1562 | 1098 | 4 | 365 | 3.6e-119 | CCT |
| *SiCMF5* | 3 (17.14) | + | *Si024852m.g* | 1156 | 960 | 3 | 319 | 7.6e-95 | CCT |
| *SiCMF6* | 3 (8.02) | - | *Si022659m.g* | 3313 | 978 | 4 | 325 | 4.0e-155 | CCT |
| *SiCMF7* | 4 (36.72) | - | *Si006393m.g* | 2874 | 1380 | 3 | 459 | 4.4e-68 | CCT |
| *SiCMF8* | 9 (1.03) | - | *Si039184m.g* | 1964 | 747 | 2 | 248 | 6.7e-38 | CCT |
| *SiCMF9* | 6 (7.34) | + | *Si014122m.g* | 2043 | 621 | 2 | 206 | 5.4e-103 | CCT |
| *SiCMF10* | 9 (17.25) | - | *Si035901m.g* | 1632 | 1239 | 5 | 412 | 5.1e-86 | CCT |
| *SiCMF11* | (37.30) | + | *Si036910m.g* | 2658 | 861 | 2 | 286 | 3.8e-79 | CCT |
| *SiCMF12* | 8 (0.82) | + | Si027626m.g | 805 | 711 | 2 | 236 | 6.6e-24 | CCT |
| *SiCMF13*^e^ | N/A | N/A | N/A | N/A | N/A | N/A | N/A | N/A | N/A |
| *SiCMF14* | N/A | N/A | N/A | N/A | N/A | N/A | N/A | N/A | N/A |
|  |  |  |  |  |  |  |  |  |  |
|  |  |  |  |  |  |  |  |  |  |
| ***H. vulgare*** |  |  |  |  |  |  |  |  |  |
| *HvCMF1* | 3HL | N/A | c_44916 | 1126 | 1026 | 2 | 341^P^ | 7.0e-73 | CCT |
| *HvCMF3* | 6HS | N/A | c_55136 | 2545 | 1086 | 2 | 361 ^P^ | e=0 | CCT |
| *HvCMF4* | 4H | N/A | AK375853 | 3585 | 1077 | 4 | 358 | e=0 | CCT |
| *HvCMF5* | 1H | N/A | c_2164420 | 755 | 543 | 3 | 180 | 8.0e-84 | CCT |
| *HvCMF6a* | N/A | N/A | AK250075 | N/A | 1035 | N/A | 344 | 1.0e-142 | CCT |
| *HvCMF6b* | N/A | N/A | AK355694 | N/A | 987 | N/A | 328 | 9.0e-138 | CCT |
| *HvCMF7* | 7HL | N/A | c_123017 | 2115 | 783 | 3 | 260 | 8.0e-61 | CCT |
| *HvCMF10* | 1H | N/A | c_1016617 | 2211 | 1224 | 5 | 408 | 1.0e-85 | CCT |
| *HvCMF11 (HvCO9)* | 1HL | N/A | AB592331 | 1969 | 888 | 2 | 295 | 4.0e-77 | CCT |
| *HvCMF13* | 5HL | N/A | c_120735 | 1065 | 738 | 3 | 245 | 1.0e-124 | CCT |
|  |  |  |  |  |  |  |  |  |  |
