# Supplementary material for: Genome Dynamics Explain the Evolution of Flowering Time CCT Domain Gene Families in the Poaceae
Source: PLoS One. 2012 Sep 24;7(9):e45307. doi: 10.1371/journal.pone.0045307 (PMC3454399; doi:10.1371/journal.pone.0045307)
Supplement: Table S5 — Poaceae ZCCT genes investigated in this study. a E-values are relative to ZCCT1 CDS. b ZF = zinc-finger domain. c partial cDNA sequence only available. N/A = not applicable. (DOCX) [file pone.0045307.s009.docx]

|  |  |  |  |  |  |  |  |  |
| --- | --- | --- | --- | --- | --- | --- | --- | --- |
| **Gene (synonym)** | **Chr** | **GenBank accession** | **Genomic (bp)** | **cDNA**  **(bp)** | **Exo-ns** | **Protein (aa)** | **e-value (% identity)^a^** | **Protein domains^b^** |
|  |  |  |  |  |  |  |  |  |
| ***T. monococcum*** |  |  |  |  |  |  |  |  |
| *VRN2* (*ZCCT1*) | 5A^m^ | AY485644 | 1896 | 639 | 2 | 213 | N/A | ZF, CCT |
| *ZCCT2* | 5A^m^ | AY485644 | 1731 | 636 | 2 | 212 | e=0 | ZF, CCT |
|  |  |  |  |  |  |  |  |  |
|  |  |  |  |  |  |  |  |  |
| ***H. vulgare*** |  |  |  |  |  |  |  |  |
| *HvZCCT-Ha* (*ZCCT-Ha*) | 4H | ABF50041 | 1669 | 645 | 2 | 215 | e=0 | ZF, CCT |
| *HvZCCT-Hb* (*ZCCT-Hb*) | 4H | DQ492698 | 1653 | 645 | 2 | 215 | e=0 | ZF, CCT |
| *HvZCCT-Hc* (*ZCCT-Hc*) | 4H | AY687931 | N/A | 264^c^ | N/A | 21^c^ | 2.0e-90 ^c^ | CCT ^c^ |
|  |  |  |  |  |  |  |  |  |
