# Supplementary material for: Genome Dynamics Explain the Evolution of Flowering Time CCT Domain Gene Families in the Poaceae
Source: PLoS One. 2012 Sep 24;7(9):e45307. doi: 10.1371/journal.pone.0045307 (PMC3454399; doi:10.1371/journal.pone.0045307)
Supplement: Text S1 — Positioning of previously mapped CMF, COL and PRR genes within the barley consensus genetic map. (DOCX) [file pone.0045307.s011.docx]

**Text S1.** Positioning of ten previously mapped *CMF, COL* and *PRR* genes within the barley consensus genetic map [47], based on standard barley-barley and barley-rice colinearity. *HvCOL* genes were mapped by [13].

**1H**

*HvCMF11* (orthologous to Os10g41100, previously named *HvCO9*): previously mapped to chromosome 1H between barley orthologues of Os10g40780 and Os10g41190 [33]. Positioned at 59.7 cM in the consensus map using marker 11_21333 (Os10g41360).

**2H**

*HvPRR37* (*PPD-H1,* orthologous to Os07g49460): previously map-based cloned to chromosome 1H [18]. Positioned at 27.3 cM in the consensus map using marker 11_21015 (Os07g49230).

*HvCO4* (orthologous to *OsC,* Os04g42020): previously mapped to the long arm of 2H between markers psr571 and mwg865. Positioned at 73.0 cM, based on marker 11_20528 (Os04g42090).

**5H**

*HvCO3* (orthologous to *OsB*, Os09g06464): previously mapped to the centromeric region of 5H between markers psb134 and wg530. Positioned at 59.4 cM, based on marker 11_20283 (Os09g07510).

**6H**

*HvCO7* (orthologous to *OsF,* Os02g08150): previously mapped to the short arm of 6H between markers psr966 and *HvCO5* (Os02g39710). Positioned at 53.3 cM, based on marker 11_21473 (Os02g08100).

*HvCO5* (orthologous to *OsD*, Os02g39710): previously mapped to the short arm of 6H between markers *HvCO7* (Os02g08150) and Psr88b. Positioned at 55.7 cM, based on marker 11_10323 (Os02g38810).

*HvCO2* (most homologous to *OsA,* Os06g16370. Note: not orthologous): previously mapped to the centromeric region of 6H between markers psr312 and psr149. Positioned at 70.0 cM, based on marker 11_20620 (Os02g49530). Note, although a rice orthologue of *HvCO2* is missing in rice, it is predicted to have resided between Os02g49580 and Os02g49590, based on intra-specific rice genome duplication [19].

**7H**

*HvCO1* (orthologous to *OsA,* Os06g16370): previously mapped to the short arm of 7H, cosegregating with marker psr105. Positioned at 73.75 cM, based on marker 11_10299 (Os06g15990).

*HvCO8* (orthologous to *OsG ,* Os08g15050): previously mapped to the short arm of 7H, distal to mwg89. Positioned at 79.6 cM (within the highly non-recombining regions associated with the centromere), based on marker 11_21302 (Os08g08220).

*HvCO6* (orthologous to *OsE,* Os06g44450): previously mapped to the long arm of 7H between markers cdo583d and mwg539. Positioned at 133.8 cM, based on marker 11_10861 (*HvCO6*).
