# Supplementary material for: Genome Dynamics Explain the Evolution of Flowering Time CCT Domain Gene Families in the Poaceae
Source: PLoS One. 2012 Sep 24;7(9):e45307. doi: 10.1371/journal.pone.0045307 (PMC3454399; doi:10.1371/journal.pone.0045307)
Supplement: Text S2 — Genetic mapping of barley genes. (DOCX) [file pone.0045307.s012.docx]

**Text S2. Genetic mapping of barley *COL* and *PRR* genes**

The non-synonymous single nucleotide polymorphism (SNP) G68/T (Ala 🡪 Ser) allowed mapping of *HvCO10* to the short arm of chromosome 4H, where it cosegregated with four SNPs at 32.5 cM. Genetic mapping using SNP G93/A (Ser 🡪 Asn) found *HvCO11* to map to the long arm of 6H at 93.1 cM, cosegregating with six SNP markers. *HvCO12* (A285/G) and *HvM* (G300/T) cosegregated with an additional twelve genetic markers at 74.1 cM on chromosome 7H. Sequencing a 3,850 bp genomic region spanning *HvCO14* identified a single SNP (C3842/T) downstream of the stop codon, allowing mapping to the long arm of 6H, between SNP markers 11_20468 and 11_10596 at 89.7 cM. *HvCO15* mapped within the highly non-recombining region of chromosome 7H predicted to contain the centromere. Similarly, using the intronic SNP A128/G, *HvCO16* was mapped to the region of the 4H genetic map within which the centromere is predicted to reside (54.4 cM). Lastly, *HvCO18* (C640/G, Pro 🡪 Ala) was found to cosegregate with fourteen genetic markers and the morphological locus *Pox* at 55.2 cM on the short arm of 2H. Genetic mapping of barley *PRR* genes finds *HvPRR59* and *HvPRR73* to map to the long arm of chromosome 4H at 52.3 and 54.4 cM, respectively. *HvPRR95* (T983/A, Arg 🡪 Ala) was mapped to 131.5 cM on the long arm of chromosome 5H, while *HvTOC1* (G936/T) was mapped to the long arm of chromosome 6H (68.5 cM).
