# Supplementary material for: Genome Dynamics Explain the Evolution of Flowering Time CCT Domain Gene Families in the Poaceae
Source: PLoS One. 2012 Sep 24;7(9):e45307. doi: 10.1371/journal.pone.0045307 (PMC3454399; doi:10.1371/journal.pone.0045307)
Supplement: Text S3 — Integration of genes genetically mapped in the OWB population into the barley consensus genetic map. (DOCX) [file pone.0045307.s013.docx]

**Text S3. Integration of *CMF, COL* and *PRR* genes genetically mapped in the OWB population into the barley consensus genetic map [47].**

**1H**

*HvCMF10* (orthologous to Os10g32900): 50.6 cM, based on cosegregation with common marker 11_20757 (Os10g32880).

*HvCMF5* (orthologous to Os05g38990): 88.2 cM, between common markers 11_10466 and 11_20475, positioned using 11_10830 (Os05g38140).

*HvCCT6a/HvCCT6b* (orthologous to Os05g51600): 138.9 cM, based on cosegregation with common marker 11_20840, positioned using marker 11_20509 (Os05g51530).

**2H**

*HvCO18* (orthologous to Os07g47140): 39.1 cM, based on cosegregation with common marker 11_109198 (Os07g47420)

**3H**

*HvCMF1* (orthologous to Os01g61900): 114.0 cM, between common markers 11_21161 (Os01g62260) and 11_21277 (Os01g61890), positioned using marker 11_21277.

**4H**

*HvCO10* (orthologous to Os03g50310): 26.2 cM, based on cosegregation with common marker 11_21418 (Os03g50290).

*HvPRR59* (orthologous to Os11g05930): 48.5 cM, based on cosegregation with common marker 11_20853 (Os11g05990).

*HvCO16* (orthologous to Os03g22770): 51.3 cM, based on cosegregation with common marker 11_20496 (Os03g21950).

*HvPRR73* (orthologous to Os03g17570): 55.6 cM, based on cosegregation with common marker 11_10262 (Os03g17470).

*HvCMF4* (orthologous to Os03g04620): 96.6 cM, based on common flanking markers 11_20454 (Os03g03720) and 11_10712, but positioned by marker 11_20838 (Os03g04340).

*HvZCCT-Ha/b/c* (not present in rice): 119.1 cM, based on cosegregation of the previously mapped *VRN-H2* locus with common marker 11_10610 (Os03g01530).

**5H**

*HvCMF13* (orthologous to Os12g01080): 56.8 cM, between common markers 11_20396 (Os12g01680) and 11_21536 (Os12g01530), positioned using marker 11_21536.

*HvPRR95* (orthologous to Os09g36220): 110.3 cM, between common markers 11_20805 (Os09g35810) and 11_11341, positioned using marker 11_20805.

**6H**

*HvCMF3* (orthologous to Os02g05470): 44.8 cM, based on cosegregation with common marker 11_10355 (Os02g54160).

*HvTOC1* (orthologous to Os02g40510): 55.9 cM, based on cosegregation with common marker 11_10513 (Os02g40514).

*HvCO14* (orthologous to Os02g49239): 70.0 cM, based on cosegregation with common marker 11_20620 (Os02g49530).

*HvCO11* (orthologous to Os02g49880): 71.1 cM, based on cosegregation with common marker 11_20577 (Os02g49800).

**7H**

*HvCO12* (orthologous to Os06g15330) and *HvM* (Os06g19444): 73.8 cM, proximal to common marker 11_20885, positioned using cosegregating markers 11_10299 (Os06g15990) and 11_11122 (Os06g21380).

*HvCO15* (orthologous to Os08g42430): 77.9 cM, based on cosegregation with common marker 11_20879 (Os08g42400).

*HvCMF7* (orthologous to Os06g48610): 114.6 cM, based on cosegregation with common marker 11_20103 (Os06g48600).
