# Supplementary material for: Genome Dynamics Explain the Evolution of Flowering Time CCT Domain Gene Families in the Poaceae
Source: PLoS One. 2012 Sep 24;7(9):e45307. doi: 10.1371/journal.pone.0045307 (PMC3454399; doi:10.1371/journal.pone.0045307)
Supplement: Text S4 — Comparative barley-rice analysis of mapped HvCMF, HvCOL and HvPRR genes. (DOCX) [file pone.0045307.s014.docx]

**Text S4. Comparative barley-rice analysis of mapped *HvCMF, HvCOL, HvPRR* and *HvZCCT* genes.**

Three *HvCMF* genes were mapped to chromosome 1H: *HvCMF10* is located in the highly non-recombining region colinear with a region of rice chromosome Os10, *HvCMF5* is located within a local inversion relative to the trend of colinearity between the long arm of chromosomes 1H and Os05, while the cosegregating genes *HvCM6a/HvCMF6b* reside close to the long arm telomeres in both species. On chromosome 2H, *HvCO18* mapped to a region of the short arm colinear with rice Os07, while on the long arm of chromosome 3H (colinear with rice Os01), *HvCMF1* is located near the end of a local inter-specific chromosomal inversion. Six genes were mapped to chromosome 4H, which is broadly colinear with rice Os03. The short arm of 4H is colinear with the distal end of rice chromosome Os03, within which *HvCO10* resides. The highly non-recombining region of chromosome 4H within which the centromere is predicted to reside coincides with a break in 4H-Os03 colinearity, spanning ~10 Mbp in rice. *HvPRR59* maps within this region, and although this gene is homologous to the rice Os03 gene Os11g05930, the disruption of simply interpreted colinearity in this region prevents further interpretation. Unambiguous 4H-Os03 colinearity commences ~3 cM from *HvPRR59* on the long arm, where *HvCO16* resides, and continues to the end of chromosome 4H, with *HvPRR73* and *HvCMF4* both fitting in with the established run of barley-rice colinearity. The barley *VRN-H2* locus (with which the *ZCCT-Ha/-Hb/-Hc* genes cosegregate) is located ~181 cM. Although deletion of *ZCCT-Ha/-Hb/-Hc* orthologues prevents direct comparative analysis in rice, comparative genomic locations between the two species was established as previously described [33], and fits in with the overall rice-barley framework of colinearity. Two genes were mapped to chromosome 5H, which shows broad colinearity with three rice chromosomes. The short arm of 5H is colinear with rice chromosome Os12, with the genetic map position of *HvCMF13* fitting in with the surrounding pattern of colinearity, and is located close to the predicted boundary between 5H colinearity with rice chromosomes Os12 and Os09. The position of *HvPRR95* on the long arm of 5H agrees with the wider colinear relationship of that region with rice chromosome Os09. *HvCMF3, HvCO2, HvCO5, HvCO11, HvCO14* and *HvTOC1* all map to chromosome 6H at positions in line with the overall pattern of colinearity between 6H and Os02. *HvCO12* and *HvCO13* map to the short arm of chromosome 7H, fitting in with the established colinear relationship with the short arm of rice chromosome Os06. *HvCMF6* and *HvCMF7* both map to the long arm of 4H, which shows an inverted colinear relationship to the long arm of rice chromosome Os02. Finally, *HvCO8* and *HvCO15* map to the highly non-recombining region of 7H predicted to span the centromere. This region appears to be colinear with rice chromosome Os08, with the two mapped barley genes integrating with the overall pattern of colinearity.
